# Long-Term Evaluation of Inserted Nanocomposite Hydrogel-Based Phosphorescent Oxygen Biosensors: Evolution of Local Tissue Oxygen Levels and Foreign Body Response

**DOI:** 10.1021/acsabm.4c00336

**Published:** 2024-05-29

**Authors:** David Chimene, Waqas Saleem, Nichole Longbottom, Brian Ko, Ananth Soundaram Jeevarathinam, Staci Horn, Michael J. McShane

**Affiliations:** † Department of Biomedical Engineering, Texas A&M University, College Station, Texas 77843, United States; ‡ Department of Materials Science & Engineering, Texas A&M University, College Station, Texas 77843, United States; § Department of Veterinary Anatomy and Pathobiology, Texas A&M University, College Station, Texas 77843, United States

**Keywords:** biosensors, hydrogels, phosphorescence, in vivo, nanomaterials, biocompatibility

## Abstract

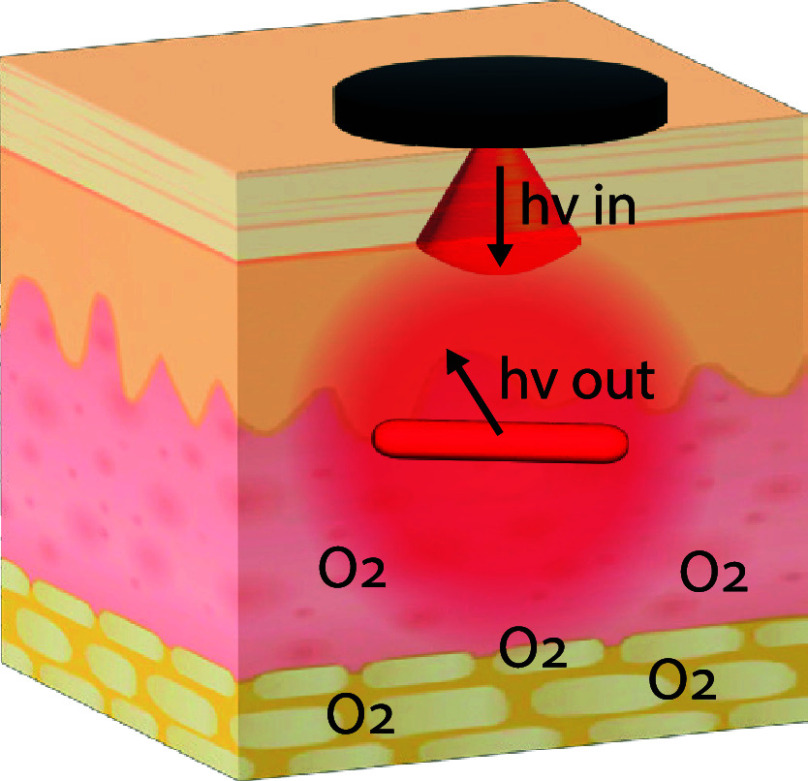

Phosphorescence-based oxygen-sensing hydrogels are a promising platform technology for an upcoming generation of insertable biosensors that are smaller, softer, and potentially more biocompatible than earlier designs. However, much remains unknown about their long-term performance and biocompatibility *in vivo*. In this paper, we design and evaluate a range of hydrogel sensors that contain oxygen-sensitive phosphors stabilized by micro- and nanocarrier systems. These devices demonstrated consistently good performance and biocompatibility in young adult rats for over three months. This study thoroughly establishes the biocompatibility and long-term suitability of phosphorescence lifetime sensors *in vivo*, providing the groundwork for expansion of this platform technology into a family of small, unobtrusive biosensors for a range of clinically relevant metabolites.

## 1 Introduction

**Figure 1:**
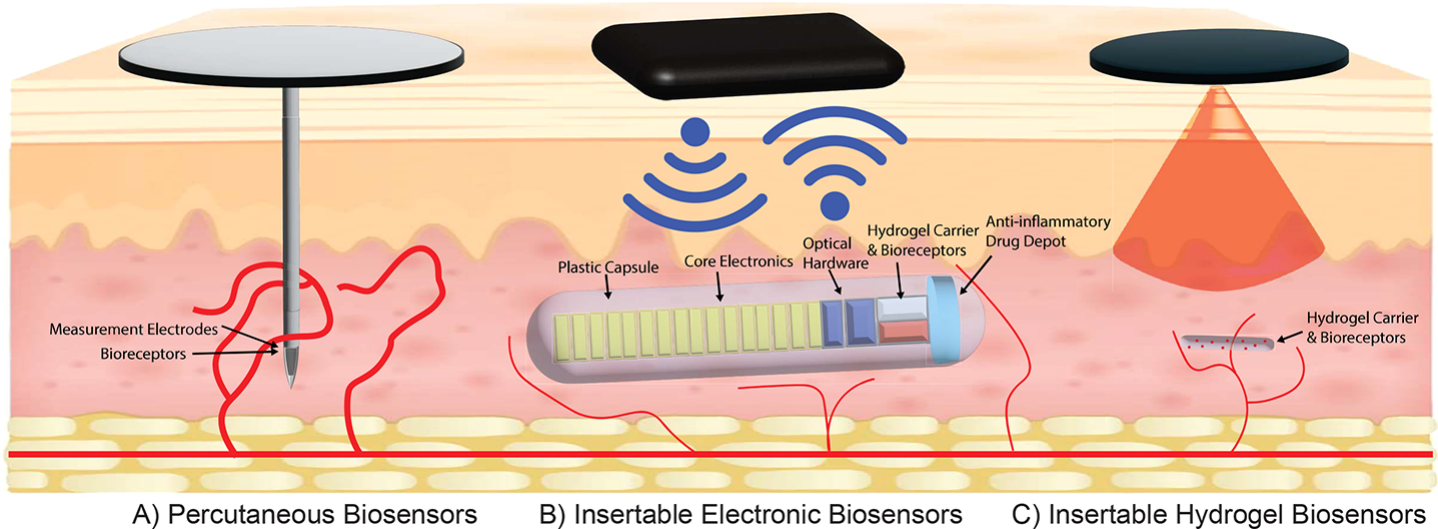
Advances in wearable biosensors. As continuous biosensors have advanced, continued innovation has enabled smaller and more biocompatible designs. (A) Percutaneous biosensors are an established technology that makes up the bulk of wearable biosensors on the market. They can be applied simply by patients and can carry biosensor signals across the skin through a physical connection. However, the device’s lifetime is limited by chronic inflammation and skin turnover. (B) Implantable biosensors were created to address these limitations. They package an optical sensor with an optoelectronic reader, an external transmitter for power and communication, and an anti-inflammatory steroid depot, all housed within a polymer capsule. These biosensors transmit signals wirelessly across the skin to circumvent the limited device lifetimes of the percutaneous biosensors. However, they are large enough to require outpatient surgery to implant and remove. C. In our insertable biosensors, optoelectronics are located in the wearable device, while the implant contains only the stabilized phosphors within a carrier hydrogel. This allows the biosensors to be much smaller and simpler to inject and simpler to manufacture. Because they can be created using a wide range of hydrogels, our insertable biosensors can be made with customizable biodegradability and minimal inflammatory response.

Biosensors, broadly defined as devices for analyzing biological processes, constitute the largest share by volume in the entire medical device industry. As of 2022, the global market size of biosensors has reached over 27 billion U.S. dollars annually.^1^ These numbers come as no surprise, considering the broad utility of biosensors in medicine and research. Biosensors provide physicians with rapid quantitative data on the biochemical status of patients. In comparison to traditional laboratory tests, which can take hours or days to get results, biosensors provide real time data for physicians to drive rapid treatment action. Likewise, researchers employ biosensors to quickly collect reams of data, allowing rapidly evolving biochemical processes to be precisely tracked in real time. For patients, portable biosensors reliably monitor their health status as they go about their daily lives. Biosensors have steadily become more portable, progressing from bulky at-home test kits to hand-held blood testers to unobtrusive wearable biosensors(Figure 1A). Wearable continuous glucose monitor (CGM) biosensors have steadily gained popularity among diabetics over the past two decades, with patients citing convenience and physicians pointing to improved patient outcomes over traditional blood sticks.^2−4^

Unfortunately, the percutaneous CGMs on the market today still require continuous perforation of the protective skin barrier, which elicits a sustained inflammatory response that causes fibrotic encapsulation until the device is removed, which limits the biosensors’ lifetime to 1 or 2 weeks.^5−8^ Additionally, the strong adhesives needed to stick the percutaneous CGMs in place frequently create skin complications. Nearly 80% of CGM wearers reported experiencing previous skin complications from the devices. When asked, 35% of CGM wearers were currently experiencing one or more skin lesions from the devices, most commonly from eczema and scarring.^9−11^

Thus, an emerging challenge in biosensing is to overcome these shortcomings by developing a new generation of fully implantable biosensors. Implantable biosensors are inserted just under the skin and wirelessly transmit data to external devices without breaking the skin barrier, effectively reducing inflammation, foreign body response, and infection risk while extending device lifetime (Figure 1B). Wearable monitors can also be moved and cleaned regularly without disrupting the biosensor itself, which is expected to reduce the skin complications associated with percutaneous CGMs.^12,13^

Leland Clark, the father of biosensors, explored the advantages of implanting biosensors under the skin as far back as the mid 1980s; however, the FDA did not approve an implanted biosensor for the commercial market until 2018. At that time, the Senseonics Eversense was approved as a glucose biosensor, combining a fluorescent glucose-binding hydrogel and an optoelectronic reader unit in a subcutaneous housing, which is inductively powered by an external wearable. Additional electronics transmit data across the skin to a reader unit, and a steroid depot is used to suppress the foreign body response.^14,15^ These pioneering devices have an extended lifetime of 3 to 6 months, and the wearable is repositionable, although they still employ adhesives. However, the Eversense requires outpatient surgical implantation and removal by a physician due to the large capsule (3.3 mm × 15 mm) needed to house its biosensing, electronic, and drug depot components. These limitations are not unique to the Eversense, however. The need for new kinds of implanted biosensors that are smaller, more flexible, more biocompatible, and even biodegradable has been increasingly recognized by the biosensing field.^3,4,16^

As fluorescence/phosphorescence sensing and biomaterials technology advances, a new generation of smaller, electronic-free biosensors known as insertable biosensors has been introduced, (Figure 1C). Insertable biosensors pack the advantages of implantable biosensors into a much smaller overall size by removing electronic components from the implant, enabling the biosensor to be entirely composed of a fluorescent or phosphorescent hydrogel. These biosensors are smaller, more flexible, easier to insert, and less expensive to manufacture. Insertable biosensors can also be made to be either recoverable or biodegradable by altering the hydrogel composition.

We have recently developed several examples of insertable nonelectronic biosensors based on phosphorescence lifetime of photoluminescent oxygen-sensing metal-porphyrin phosphors.^17,18^ In each case, a metal-porphyrin complex emits phosphorescence in response to excitation with red light, and this fluorescence is quenched in the presence of oxygen. Because the lifetime of the phosphorescence reaction is inversely related to the presence of oxygen, the phosphorescence lifetime can be used to indirectly measure the oxygen concentration within the hydrogel. Similar metal-porphyrin complexes have tantalized biosensor researchers for years, but their application has been limited by aggregation issues and a tendency to migrate away from the target site.^19,20^

Profusa, a company our lab has collaborated with on previous projects, currently markets an insertable oxygen sensor based on phosphorescence lifetime using an earlier metal-porphyrin sensor. This sensor is used for reporting tissue oxygenation in peripheral artery disease. Unlike pulse oximetry, which reports hemoglobin oxygenation within blood, insertable sensors provide a more nuanced picture of oxygen concentrations within tissue, which can differ from blood oxygenation based on microvascular functionality and changes in tissue metabolism.^21^ Therefore, tissue oxygen sensors are of keen interest for treating peripheral artery disease, ischemia and reperfusion injury, microvascular disease in diabetics, and in monitoring wound healing.^22^ We have expanded this family of metal-porphyrin insertable sensors by developing new methods to encapsulate phosphor complexes in hydrogels that prevent phosphor aggregation, improve optical efficiency and sensitivity, and reduce the amount of metal-porphyrin complex required per sensor.^23^ In particular, the use of microsphere encapsulation also allows us to tightly control nutrient diffusion to the phosphor, providing an avenue toward insertable biosensors beyond oxygen, including glucose, lactate, or amino acid biosensors. The next step is to incorporate these new sensing advances with biocompatible hydrogel carrier materials to create practical sensors and investigate their performance in vivo.

One of the primary obstacles to practical, long-lasting, insertable biosensors is engineering a favorable foreign body response. The foreign body response plays a definitive role in a biosensors safety, performance, and longevity.^24,25^ It begins with biofouling of the biomaterial surface, which is then quickly surrounded by immune cells and finally fibrotic encapsulation. Each of these steps progressively chokes off diffusion between the probe and the interstitial fluid. This is the primary limiting factor for CGM device lifetime, requiring replacement with a new probe in a different location, where the process starts again. Fortunately, improved biomaterials and device design can be used to mitigate the foreign body reaction. In this paper, we combined several biomaterials strategies to minimize the foreign body response to our sensors, including biocompatible materials, smaller and softer implants, and both biodegradable and nondegradable sensors.^8,26−29^

In this paper, we developed a comprehensive understanding of biosensor biocompatibility and long-term performance *in vivo*. We engineered soft, insertable sensors less than one-hundredth the size of commercial implantable biosensors using a selection of biocompatible hydrogels intended to minimize foreign body response and extend biosensor lifetime. By optimizing these sensors to function within living tissue and then evaluating their effectiveness for over three months *in vivo*, we assessed the potential of each sensor formulation for long-term performance. Oxygen-sensitive phosphors were either encapsulated in alginate microspheres or enmeshed in a stabilizing ethylcellulose nanofibrous network, and then dispersed in different hydrogel carriers to create the oxygen sensitive hydrogel sensors. The sensors were then inserted below the skin of healthy adult rats and monitored for more than 3 months. Regular *in vivo* testing provided data to evaluate our various sensor designs for signal strength and consistency, durability, biodegradation, and biocompatibility over an extended evaluation period. Measurement data also allowed us to assess local oxygen level variations in the tissue surrounding the inserted devices as a function of time, sensor location, and local tissue structure. Following the *in vivo* experiments, sensors and surrounding tissue were histologically evaluated for biocompatibility, biodegradation, and foreign body response.

By establishing the long-term performance and biocompatibility of our insertable oxygen sensor designs *in vivo*, this project demonstrates the practicality of our insertable sensors. We envision these sensors for clinical use monitoring tissue oxygenation in wound healing applications as well as ischemia and reperfusion injuries, peripheral artery disease, and microvascular disease in diabetics. Biodegradable formulations will also allow sensors to be removed by the body over time instead of requiring a removal procedure. Finally, this technology also advances a platform technology that can be used as the basis for an array of insertable metabolite biosensors, including glucose and lactate. These may be realized by combining analyte-specific enzymes with the phosphorescent oxygen reporters. Thus, we envision creating insertable biosensors for a range of conditions and expect the advantages of our approach to improve the lives of diabetics by providing a low-cost, less disruptive option for managing treatment.

## 2 Materials and Procedures

In this article, we evaluated the long-term performance and biocompatibility of our phosphorescence lifetime-based oxygen sensors *in vivo*. We prepared oxygen sensitive hydrogels with an injectable form factor about the size of a rice grain (0.5 mm × 0.5 mm × 5 mm), characterized their performance *in vitro* and *ex vivo*, and then inserted the sensors into healthy adult rats to evaluate long-term functionality and biocompatibility under realistic conditions. Finally, we undertook a comprehensive histological evaluation of the recovered sensors and surrounding tissue.

### 2.1 Phosphors

Two similar oxygen-sensitive phosphors were employed in these experiments: palladium(II) meso-tetra-(sulfophenyl) tetrabenzoporphyrin sodium salt phosphor, nicknamed “HULK” for its strength and bright green color, and palladium(II) meso-tetra(4-carboxyphenyl)tetrabenzo-porphyrin) (PdBP) phosphor. These two phosphors were chosen for this experiment for several reasons. Both PdBP and HULK have been used successfully in previous sensor embodiments, and have exhibited excellent stability, sensitivity, and wide dynamic range.^23,30^ PdBP-encapsulating ethylcellulose nanofibers are also cytocompatible with 3T3 fibroblast cells at the phosphor concentrations used in these sensors.^23^ Metalloporphyrin phosphors have also been successfully coencapsulated with enzymes to measure other biologically relevant small molecules including glucose and lactate *in vitro*, making this phosphor group an attractive match for future investigation into other nutrient sensing *in vivo* biosensors. With this in mind, we elected to evaluate both the HULK-in-microspheres and PdBP-in-ethycellulose forms of the metalloporphyrin based oxygen sensors.

Both porphyrin phosphors have long phosphorescence lifetimes that last hundreds of microseconds and can be measured free of background scattering from biological tissues. This is due to the temporal separation between signals generated from target-phosphor interaction (on the order of microseconds) versus natural luminescence lifetime (on the order of nanoseconds) arising from the surrounding proteins. The difference between the two metallobenzoporphyrin molecules is the functional end groups (sulfonic acid versus carboxylic acid), which confer slightly different acidity and solubility that affects aggregation and other environmental interactions (e.g., with surrounding matrix). PdBP disperses more effectively into the hydrophobic ethylcellulose matrix, so it was selected over HULK for the nanoethylcellulose carrier gels.

### 2.2 Phosphor Micro/Nano Carrier Strategies

**Figure 2:**
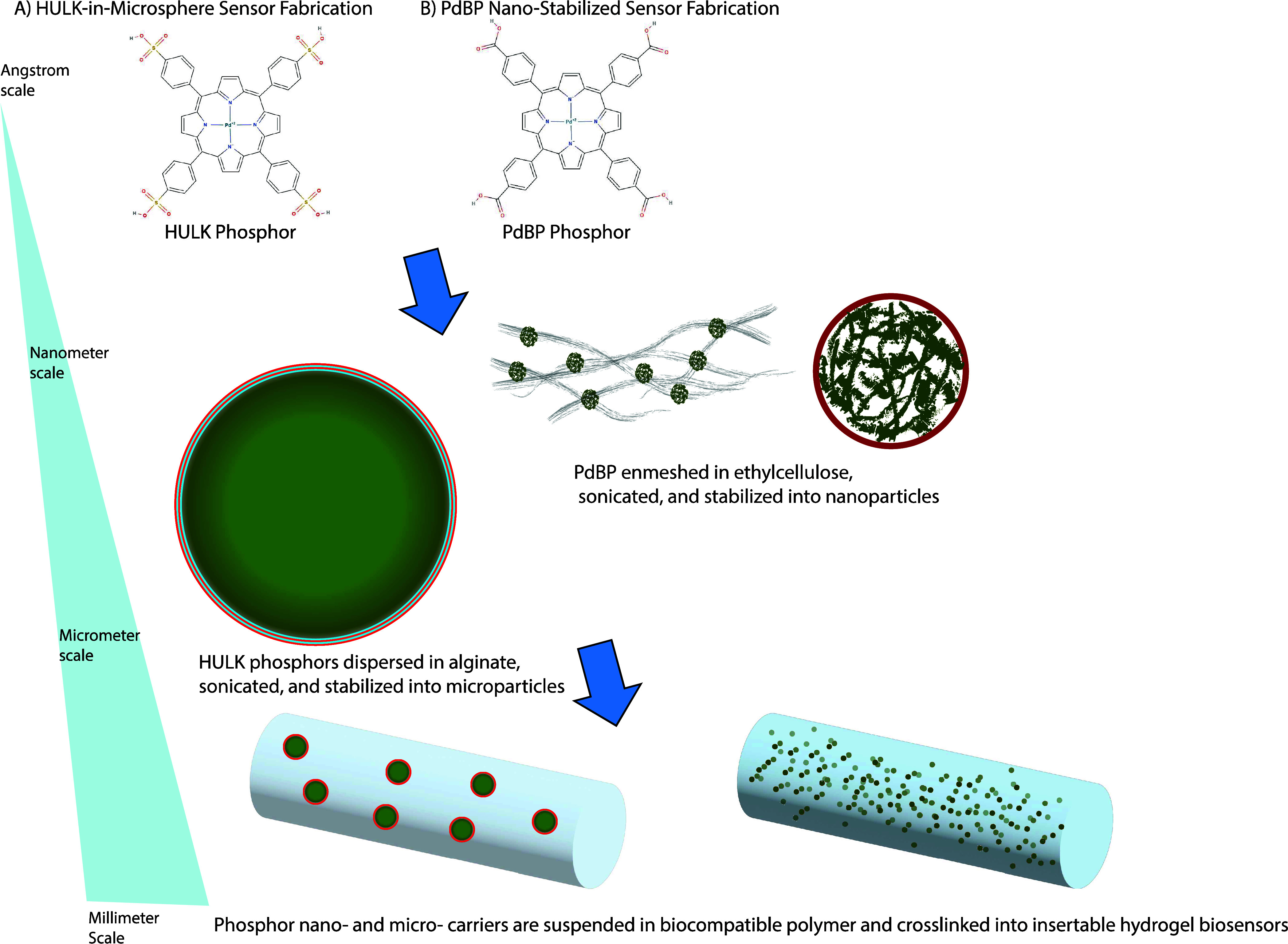
Fabrication of (A) HULK-in-microsphere sensors and (B) PdBP nanostabilized sensors. Top: Two similar oxygen-sensitive metalloporphyrin phosphors, HULK and PdBP, were incorporated into the sensors using distinct strategies. Middle: Hulk phosphors were cast into alginate microspheres, which were laminated with alternating charged layers of polyelectrolytes to contain the phosphors while allowing oxygen to diffuse freely. The less polar PdBP phosphors were trapped into a nanoethylcellulose matrix and stabilized with surfactant, which entangled and stabilized the phosphors while also allowing free access to oxygen. Bottom: Both HULK microspheres and PdBP nanoethylcellulose could be easily mixed into any of our available hydrogel solutions and cross-linked into an insertable sensor. Empty microspheres were also incorporated into PdBP-nanoethylcellulose designs in order to increase light diffusion.

The metalloporphyrin phosphors were incorporated into our hydrogel sensors in two distinct forms: as HULK-impregnated alginate microspheres surrounded by successive charged bilayers of positive polystyrenesulfonate (PSS) and negative poly(allylamine hydrochloride) (PAH) or as PdBP stabilized and entangled within an ethylcellulose nanofibrous network (Figure 2). These two approaches were both developed to prevent phosphor aggregation, which would otherwise reduce the sensitivity and effective signal strength. Each approach has distinct advantages and limitations, as discussed below.

The HULK-in-microsphere approach was designed as a way to concentrate the HULK phosphor within functional alginate microsphere units that can be integrated into a wide range of hydrogel carriers (Figure 2A) and could also easily host enzymes in future experiments. These microspheres were stabilized by successive alternating charged layers of polyelectrolytes (PSS and PAH), which were deposited using alternating charge saturation to form a nanoscale coating that creates a stable “microbubble” that envelops the HULK, preventing it from diffusing away. Another key feature of this format is that the nanocoatings can also be used to tune the diffusion rates of small molecules, such as glucose or lactate, into the microsphere. When the microspheres contain both HULK phosphors and, for example, glucose oxidase, this feature can be used to match glucose diffusion into the microsphere with glucose oxidase reaction kinetics. This effectively pairs ambient glucose levels with oxygen concentration within the microspheres, creating a glucose biosensor. This general technique has been demonstrated *in vitro* in our previous papers for glucose and lactate, and is likewise expected to be applicable to other oxygen-depleting enzyme substrates as well, such as uric acid. This makes these microspheres a versatile target for future development toward additional *in vivo* applications.^23,31−33^

In contrast to the microsphere-only approach, our other sensing system was based on using PdBP phosphors stabilized in a mesh of ethylcellulose nanofibers (Figure 2B). A recent study has shown that these nanofibrous networks can stabilize high concentrations of PdBP phosphors, preventing the hydrophobic PdBP phosphors from aggregating when exposed to a hydrophilic aqueous environment. This effect improves the sensor’s efficiency by ensuring that each phosphor consistently interacts with available oxygen.^23^

### 2.3 Oxygen-Sensitive Microparticle Synthesis

The first step for creating HULK-in-microsphere insertable sensors was creating alginate microparticles. A 5 mL alginate solution at 3% weight per volume (w/v) in water (sodium alginate (low viscosity, 250 cps, Mw 12 k-80 k, Sigma-Aldrich) was mixed with 500 μL of DMSO (dimethyl sulfoxide) containing 10 mM HULK (Palladium(II) meso-tetra-(sulfophenyl) tetrabenzoporphyrin sodium salt) (MW: 1327.55g/mol, excitation: 630 nm, Emission: 800 nm) (Frontier Scientific). The solution was then gently crushed and completely dissolved overnight.

Next, 260 uL of SPAN 85 (Sorbitan Trioleate, TCI) was mixed into a 10.8 mL iso-octane solution (2,2,4-trimethylpentane, J.T. Baker) in a 50 mL centrifuge tube. The dissolved alginate solution and the iso-octane solution were then mixed to form an emulsion using an IKA T25 Easy Clean Homogenizer at 8000 rpm for 10 s. Next, a mixture of 130 μL of TWEEN 85 (polyoxyethylene sorbitan trioleate, TCI) in 1.5 mL of iso-octane was added into the alginate emulsion and homogenized for 10 s. Then, we added 4 mL of 10% weight per volume (w/v) CaCl2 in DI water into the emulsion and mixed for 15 s. The emulsion was poured into a 500 mL round-bottom flask with a magnetic stir bar and spun for 20 min at 300 rpm at room temperature to mix calcium chloride uniformly into the emulsion. The emulsion was transferred into a 50 mL centrifuge tube and centrifuged at 626*g* for 2 min. The supernatant was removed, and the pellet was resuspended in 1 mL of deionized water (DIH2O). The solution was recentrifuged at 626*g* for 1 min, the supernatant was again discarded, and 1 mL of DIH2O was added. This centrifugation and disposal process above was then repeated again but with a PSS Wash instead of DIH2O in order to separate the iso-octane from the aqueous solution. The iso-octane was discarded and the 5–6 mL final alginate suspension was transferred into 2 mL centrifuge tubes, then the microparticles were subjected to polyelectrolyte solutions for successive deposition of nanofilm coatings of alternating charges through the layer-by-layer self-assembly procedure described below.^34^

#### 2.3.1 Microparticle Coating via Layer-by-layer Self-Assembly

To create a single bilayer, the alginate microparticles were sequentially soaked in PAH, PAH wash buffer, PSS, and then PSS wash buffer as follows. The alginate microparticle suspension was centrifuged at 2000*g* for 2 min, and the supernatant was replaced with 1 mL of PAH, pipet mixed, recentrifuged, and resuspended in the PAH wash buffer. The centrifugation was then repeated, and the supernatant was replaced with 1 mL of PSS, then recentrifuged and replaced with 1 mL of PSS wash buffer. These steps completed a single bilayer. This procedure (PAH, PAH wash, PSS, PSS wash) was repeated 5 times to get a total of 5 bilayers. The final PSS wash was repeated one extra time. All tubes were recombined into a 5 mL centrifuge tube and were left in the final PSS wash. They were stored at 4 °C and have been tested to be shelf stable for at least one year.

#### 2.3.2 Microparticle Characterization

Following the completion of the requisite bilayer steps, a volume of 30 μL of alginate microparticle suspension, in triplicate, was transferred into vacant 0.5 mL centrifuge tubes. These tubes were dried overnight in a vacuum oven. The dried microparticles were weighed to calculate the concentration of dried microparticles in the original stock suspension. Size distribution of alginate microparticles was analyzed via Nexcelom Cellometer Mini that reported the mean diameter size of microparticles around 9.8 μm and least number of aggregates within the stock suspension (Figure S1A,B).

#### 2.3.3 Oxygen Sensitive Nanoparticle Suspension Synthesis

The oxygen sensitive nanoparticles were prepared as a nanoemulsion. 100 mg of ethylcellulose was dissolved in 5 mL of tetrahydrofuran by stirring overnight. 2 mg of PdBP was then sonicated into the solution for 30 min, and then the solution was filtered through a 0.2 μm PTFE syringe filter into a glass vial. In a separate 50 mL flask, 100 mg of surfactant was dissolved in 20 mL of deionized water by using a probe sonicator in an ice bath. Then, the THF-phosphor-polymer solution was quickly injected into the surfactant–water solution, and sonication continued for another 2 min.

The resulting bright green nanoparticle suspension was then filtered through a 100 μm nylon filter and then concentrated down to 3 mL via centrifuge filtration (3500 RMP, MWCO 100 KDA). The suspension was then quintuple washed with nanopure water to remove excess surfactant. The prepared nanoparticle suspension was stored at a concentration of 12.6 mg/mL at room temperature in a dark container.

### 2.4 Hydrogel Carriers

In this study, we aimed to directly evaluate the long-term *in vivo* performance of these oxygen sensitive sensors. A primary concern for implanted biosensors is biocompatibility and the foreign body response. Therefore, we elected four different promising biocompatible hydrogel matrices to investigate for performance in a living animal model: 1.5% alginate, 20% gelatin-alginate-collagen (GAC), 20% bovine serum albumin (BSA), and 20% poly [2-(methacryloyloxy) ethyl]phosphorylcholine (MPC). Alginate was chosen for its long track record as a stable and biocompatible ionically cross-linked hydrogel and because it has been used successfully in previous development efforts with excellent in performance.^32^ By combining alginate with gelatin and collagen, previous studies have shown improved cell adhesion and tissue integration with the host tissue over alginate alone. Bovine serum albumin (BSA) hydrogels are widely popular as protein-based hydrogels and have been used extensively *in vivo* with excellent results in terms of biocompatibility and biodegradability.^35^ MPC has been gaining attention in recent years because its neutral zwitterionic structure has yielded good biocompatibility and an antifouling effect that may improve biosensor longevity *in vivo*.^36^

#### 2.4.1 Optimizations and Verification of Sensor Design

Sensor geometry was optimized to create hydrogel sensors suitable for use *in vivo*. Each sensor was designed to be 1.25 μL in volume (0.5 mm × 0.5 mm × 5 mm and contained less than 122 ng of HULK phosphor (within microparticles) or 38 ng of PdBP (bound to ethylcellulose nanoparticles). Initial sensor geometries were calculated using modeling performed in our lab, which indicated that at the above concentrations, a cylinder 500 μm in diameter and 5 mm in length would be sufficient to reliably detect the phosphorescent lifetime while still allowing for consistent oxygen concentrations throughout the inserted sensor. Sample images from each sensor type can be seen in Figure S1C. The cross section of the sensor was changed from round to square for easier manufacturing on such a small scale.^4,37^

#### 2.4.2 Incorporating Phosphors and Carriers into Hydrogel Sensors

To create the 4 HULK-in-microsphere based insertable sensors, the oxygen-sensing alginate microparticles were dispersed into the four different types of hydrogels (Figure 2a). Each hydrogel type contained 8.8 mg of dry weight equivalent alginate microparticles dispersed in a 400 μL mold, from which the insertable sensors were then cut. We separately prepared the 2 nanoethylcellulose other sets of hydrogels that incorporated PdBP phosphors stabilized in ethylcellulose nanofibers (Figure 2b). In these cases, 1.5% alginate and 20% MPC gels were loaded with 0.75 mg/mL ethylcellulose nanoparticles with phosphors. Note that after cross-linking, different hydrogels swell or contract slightly in Tris buffer solution, requiring slightly different prepared concentrations to arrive at the same final dimensions and microsphere concentrations.

#### 2.4.3 Alginate Hydrogel Sensors (HULK-in-Microsphere)

The initial step for alginate hydrogel synthesis was to add 200 μL of 3% w/v alginate in a 2 mL centrifuge tube. Then, we added 75 μL of 8.8 mg of alginate microparticles to the centrifuge tube. After the alginate microparticles were added, we added 25 μL of aqueous CaCO_3_ (33 mg/mL) cross-linker solution to the mixture and vortexed. Then, we added 100 μL of 50 mM MES buffer (pH 6.1) to free the calcium ions, activating the cross-linker solution. The solution was pipetted into a template made from two glass slides sandwiched by a 0.75 mm Teflon spacer. The solution was then set aside to allow the hydrogel to cross-link for 15 min. After initial cross-linking process, the hydrogel was removed from the template and stored in 10 mM TRIS, pH 7.2, with 10 mM CaCl2 (PSS wash).

Finally, alginate hydrogels are soaked in tris buffer 10 mg with 10 mg CaCl2 overnight as a final cross-linking step. This also slightly shrinks the hydrogels.

#### 2.4.4 Alginate Hydrogel Sensors (PdBP-in-Nanoethylcellulose)

For the alginate hydrogel using nanoparticles, a half concentration (4.4 mg) of blank microparticles without phosphors was included as a scattering agent. Accordingly, 4.4 mg of alginate particles were triple rinsed in DI water. Next, 75 μL of PdBP-ethyl cellulose nanoparticle suspension (4 mg/mL) was added to the mixture followed by 200 μL of a 3.0% aqueous solution of sodium alginate and 25 μL of 33.3% aqueous suspension of calcium carbonate suspension in deionized water. After mixing the above mixture thoroughly, 100 μL of MES buffer at pH 6.1 was added, and the whole mixture was quickly transferred into a 0.75 mm template. Hydrogels were finally stored in 10 mmol of Tris buffer with 1 mmol of CaCl_2_ overnight.

#### 2.4.5 Gelatin-Alginate-Collagen Hydrogel Sensors (HULK-in-Microsphere)

The synthesis of Gelatin-Alginate-Collagen (GAC) hydrogels began with preparing 1 mL of 25% Gelatin (Porcine Type A, VWR) solution in MES (pH 6.1) buffer. The solution was then heated to 60 °C. Next, 1 mL of 5.25% alginate (alginic acid sodium salt, Sigma-Aldrich) solution in MES (pH 6.1) buffer is prepared and heated to 60 °C. The solution was allowed to cool to 37 °C in a water bath. 5.23 mg of solid *N*-hydroxysuccinimide (NHS) (TCI Chemicals) powder was then added to the alginate solution. The collagen (Rat Tail-Type I, Advanced Biomatrix, 4 mg/mL) solution was neutralized with the provided acid solution, as per the vendor’s recommended procedure. 160 μL of 25% gelatin solution, 76.2 μL of 5.25% alginate solution (premixed with 5.23 mg/mL of NHS), and 88.90 μL of neutralized collagen solution (4 mg/mL) were mixed in a scintillation vial with a small magnetic stirrer, and the resulting solution was quickly vortexed while holding the temperature at 37 °C in the water bath. The final concentration of each component in gels was around 10% gelatin, 1% alginate, and 9% collagen. We then washed 8.8 mg of alginate microparticles (oxygen or glucose sensing) with DI water three times and resuspended in ∼75 μL volume of DI water in 2 mL centrifuge tube. The GAC solution was then pulled into a 1 cm^3^ syringe along with 8.8 mg of alginate microparticle in 75 μL of DI water and mixed in the syringe. Finally, the mixture was injected using an 18.5-gauge needle into a 0.75 mm template as described above. The glass slides were cooled in a 4 °C refrigerator for quick gelation for 10 min and then submerged in Petri dishes filled with 10 mL of MES buffer (pH 6.1) predissolved with EDC (1-ethyl-3-(3-(dimethylamino)propyl)carbodiimide, Sigma-Aldrich) at 10 mg/mL concentration. The dish was then covered and stirred for 2 h. Finally, the gels were washed twice with DI water, covered, and stored in a 100 mM Tris-buffer with 10 mM CaCl2 (pH 7.2) at 4 °C.

#### 2.4.6 Bovine Serum Albumin(BSA) Hydrogel Sensors (HULK-in-Microsphere)

A 30% bovine serum albumin (Fraction V, Protease Free, GoldBio) stock solution was prepared in PBS (pH 7.4). Separately, in a 2 mL centrifuge tube, 267 μL of 30% BSA solution was mixed with 42.3 μL of PBS buffer solution. Concurrently, 8.8 mg of alginate microparticles in 75 μL of DI water were combined to the BSA-PBS mixture solution and mixed for 15 min. Lastly, 16 μL of glutaraldehyde (25% in water, Sigma-Aldrich) was added to the mixture, and the solution was quickly pipetted in between two glass slides that were paraffin-wrapped from interior and separated by a 0.75 mm Teflon spacer. The hydrogel was incubated at room temperature for 15 min followed by washing with 100 mM Tris buffer (with 10 mM CaCl_2_) solution, and stored at 4 °C. This resulted in a final hydrogel concentration of 20% BSA. Samples were also rinsed in glycine (1% glycine in DI water, stirred for 30 min) to ensure trace glutaraldehyde from cross-linking was inactivated prior to insertion.

#### 2.4.7 MPC Zwitterionic Hydrogel Sensors (HULK-in-Microsphere)

Zwitterionic hydrogels containing alginate microparticles were prepared by adding 8.44 mg of *N*,*N*′-methylenebis(acrylamide) (BIS) in 425 μL of a 50 mM TRIS buffer followed by the addition of 91.6 mg of 2-methacryloyloxyethylphosphorylcholine (MPC). Then, 17.6 mg of dry weight equivalent of alginate microparticles suspended in a total of 75 μL of DI water was added followed by 12 μL of a 10 mM aqueous solution of ammonium persulfate (APS) and 2 μL of *N*,*N*,*N*′,*N*′-tetramethyl ethylenediamine (TEMED). The solution was bubbled with nitrogen bubbling for 30 s and transferred into a rectangular sandwich mold with a Teflon spacer and placed in a nitrogen environment for 4 h for cross-linking. Finally, the hydrogel was washed in a 10 mM TRIS buffer solution with 10 mM calcium chloride for 12 h and stored in a fresh TRIS buffer solution at 4 °C, for a final concentration of approximately 20% MPC.

#### 2.4.8 MPC (2-Methacryloyloxyethylphosphorylcholine) Zwitterionic Hydrogels (PdBP-in-Nanoethylcellulose)

Zwitterionic hydrogels containing nanoparticles were prepared by dissolving 8 mg of *N*,*N*′-methylenebis(acrylamide) (BIS) in 406 μL of 50 mM TRIS buffer followed by the addition of 91.6 mg of 2-methacryloyloxyethylphosphorylcholine (MPC). A half concentration of blank alginate microparticles was also added to increase light scattering. Then, 94 μL of a nanoparticle suspension containing 1.56 mg of nanoparticles was added along with 12 μL of a 10 mM aqueous solution of ammonium persulfate (APS) and 2 μL of *N*,*N*,*N*′,*N*′-tetramethyl ethylenediamine (TEMED). The solution underwent 30 s of nitrogen bubbling and was then transferred into a rectangular sandwich mold with a Teflon spacer and placed in a nitrogen-rich environment for 4 h to facilitate cross-linking. Finally, the hydrogel was washed for 12 h in a 10 mM TRIS buffer solution containing 10 mM calcium chloride and stored in fresh TRIS buffer at 4 °C, resulting in a final MPC concentration of approximately 20%.

#### 2.4.9 Silica Beads – Positive Control

22 mg/mL of glass microspheres (Transfer Standard, Malvern Instruments, Size: 15–120um) was placed in a 100 mM Tris buffer with 10 mM CaCl_2_. Immediately before use, the vial was shaken to briefly resuspend the microspheres for injection.

#### 2.4.10 Sterile Saline – Negative Control

Normal saline solution was prepared by dissolving 0.9% (w/v) sodium chloride in nanopure water.

### 2.5 Electron Beam Sterilization

All samples were prepared as described and stored in a buffer solution in preparation for E-beam. All sensors and controls were E-beam irradiated at a target dosage of 25 kGy through Electron Beam (L3 Pulse Sciences, beam energy: 10 MeV, beam power: 15 kW, distance: 32 in.) at the National Center for Electron Beam Research (Texas A&M University). Postirradiation, samples were analyzed through alanine pellet dosimeter (Bruker E-Scan) to confirm the absorbed dose in the samples to be <25 kGy (Table 4).

### 2.6 Phosphorescence Lifetime Reader Devices

In order to quantify the phosphorescence lifetime generated by the sensors, a previously reported reader system was used both in vitro and in vivo. Briefly, the phosphorescent reader head determines the phosphorescence lifetime using time-domain analysis, and consists of three different channels: the excitation channel, the emission channel, and the temperature channel (Figure S2A).^38,39^

Starting in the excitation channel, an LED (Luxeon LXM2-PD01–0050, Lumiled) emits photons with an average wavelength of 632.5 nm and full width at half-maximum (fwhm) bandwidth of 50 nm. These photons pass through an excitation optical filter with a center wavelength of 631 nm and fwhm BW of 36 nm (Semrock, Mfr.#FF01–631/36), then are collimated via ball lens (Edmund Optics, Mfr.# 43–644), and exit through a 1 mm aperture onto the subject’s skin. The LED is 175 mil above the aperture; the excitation filter is 135 mil above, and the ball lens is 10 mil above the aperture.

**Figure 3:**
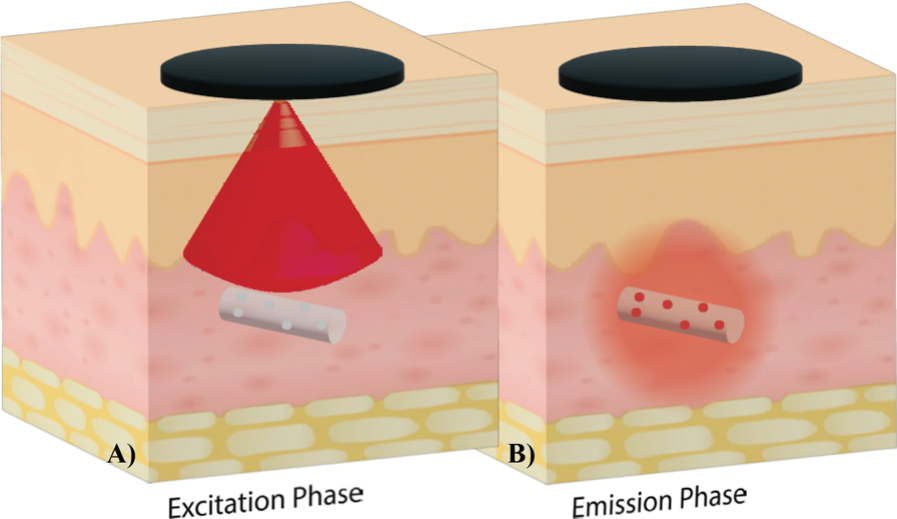
Collecting phosphorescence lifetime readings from an in vivo biosensor. In the excitation phase, the reader head emits red light to excite the biosensor’s phosphors, which begin to phosphoresce. In the emission phase, the reader head detects the infrared light emitted from the biosensor. The length of time before the phosphorescence is quenched is dependent on the oxygen interactions with the phosphors, allowing oxygen levels to be quantified. Phosphorescence lifetime measurements are unaffected by the implantation depth, which allows the optoelectronic components to remain outside the body, creating smaller and less invasive biosensors.

The excitation photons pass through the animal tissue, and some reach the phosphorescent sensor in tissue (Figure 3A). These photons elicit phosphorescence, causing the sensor to emit photons centered around 809 nm, some of which exit the tissue back toward the reader head (Figure 3B). Escaping photons are collected through a 1 mm circular emission aperture 6 mm away from the excitation aperture, center to center. A collimating lens (Thorlabs, Mfr.#354330) in the emission channel maximizes the number of the photons that continue straight up through the emission filter(centered at 809 nm wavelength and a 81 nm fwhm bandwidth) and finally hit a photodetector (SensL, C-Series) at the end of the emission channel. The reader heads use silicon photomultiplier tubes to convert photon flux to a voltage, which is collected by a data acquisition board controlled using LabVIEW. Within the custom LabVIEW code, the decay curve of the detected emission is fit to an exponential curve to determine the decay rate of the emission, referred to as the *lifetime*. This value can be approximated as the time that it takes for the phosphor signal to decay to 33% of its initial fully excited value.

Physically, the collimating lens is located 100 mil above the aperture, and the photodetector is 400 mil above the aperture. The emission photons hitting the photodetector produce an electrical current proportional to their intensity. This current is converted to a readable voltage using a transimpedance amplifier made by putting a resistor in the negative feedback of a wideband operational amplifier (Texas Instruments, Mfr.#OPA656NB/250).

The temperature aperture is 100 mil in diameter and hosts a thermistor (Littelfuse, Mfr.#KS103J2) that enables the reader head to measure the temperature in its vicinity.

### 2.7 *In Vitro* Oxygen Sensing

Tissue oxygen levels can vary widely *in vivo* due to the competing forces between oxygen delivery and consumption, with local diffusion factors also playing a key role.^21,40,41^ Therefore, oxygen sensors for tissue monitoring must be able to accurately report oxygen levels over a wide range of conditions: from 0% oxygen to atmospheric concentrations of about 21%. To test sensors performance under these varying oxygen conditions, our sensors were constantly read while subjected to a series of representative oxygen concentrations using our in-house flow cell setup (Figure S2B).^42^ These tests were performed by using matching sensors that were subjected to the same e-beam sterilization and storage conditions as those used in surgery. Each calibration is unique to the sensor type and batch.

Briefly, sensors were affixed onto a transparent acrylic sample holder using rubber cantilevers, and the sample holder was housed and sealed within a rectangular flow cell with optical readout windows through which measurements could be taken. A controlled mixture of air and nitrogen was introduced at defined ratios using digital mass flow controllers (MKS Instruments PR4000B controller) to achieve dissolved oxygen levels at 0, 25.7, 129.5, and 257.9 μM for testing. The gas mixture was bubbled into 500 mL of 100 mM TRIS buffer (with 10 mM CaCl_2_) solution filled in a round-bottomed flask, constantly stirred inside the incubator at 37 °C, and circulated through the flow cell (Figure S2B).^17,23^ Manipulation of oxygen concentrations was digitally controlled via a custom LabVIEW program and validated using an electrochemical oxygen probe (UniSense O_2_ probe). Finally, the lifetimes of individual hydrogel sensors were recorded using the 1D reader heads described above, collecting data at 10-s intervals.

These oxygen tests quantify the inverse linear relationship between the local oxygen concentration and phosphorescence lifetime (Figure S3). By establishing a relationship between the known oxygen concentration and the observed lifetime value, we created a calibration for each sensor material type for use in animal studies.

### 2.8 Ex Vivo Oxygen Sensing

To confirm that the sensors would function as designed *in vivo*, preliminary subcutaneous tests were performed on donated refrigerated Sprague–Dawley rat cadavers. The cadaver was allowed to equilibrate to room temperature, and the dorsal skin was gently separated from the underlying fascia to create a space for sensors to be tested beneath the skin. Baseline readings were first collected from sensors of each type, optically interrogated in a Petri dish under ambient oxygen conditions. The sensors were then placed beneath the skin to model different positions in living animals. Once positioned, the sensors were tested through the skin with the reader, and phosphorescence lifetime and signal-noise-ratio (SNR) values recorded. The values for readings under ambient conditions and values from ex vivo tissue were compared using a unity plot and a Bland-Altman plot (Figure S4).

### 2.9 Animal Studies

We set out to investigate the long-term *in vivo* performance of our insertable hydrogel sensor designs over an extended 3 month period followed by a thorough histopathological evaluation of each sensor and the surrounding tissue. We selected rats for this study for their hardiness and ease of handling and because they are the smallest lab animal with comparable skin thickness to man.^43^ The Sprague–Dawley breed is a healthy and immunocompetent outbred model with a deserved reputation for affability and ease of handling. Immunocompetence is a critical trait for evaluating biocompatibility, degradation, and device performance because the immune system directs the foreign body response and exerts significant control over blood flow. Both sexes were equally represented in the study to account for their different sizes and investigate any potential differences in biocompatibility and skin characteristics. This study encompasses a significant portion of the lifespan of a rat, so we enrolled adults 6 to 8 months old at the experiment’s outset in order to minimize changes in size while still remaining in the healthiest period of the rats’ life cycle. Even so, the average female rat weight increased from 270 to 340 g over the course of the experiment, while the males bulked up from 460 to 550 g. Animal studies were IACUC approved under AUP# IACUC 2021–0066 Reference Number: 137661. All 6 Sprague–Dawley rats involved were proven breeders from Envigo, and each female was verified nonpregnant prior to shipping.

**Figure 4:**
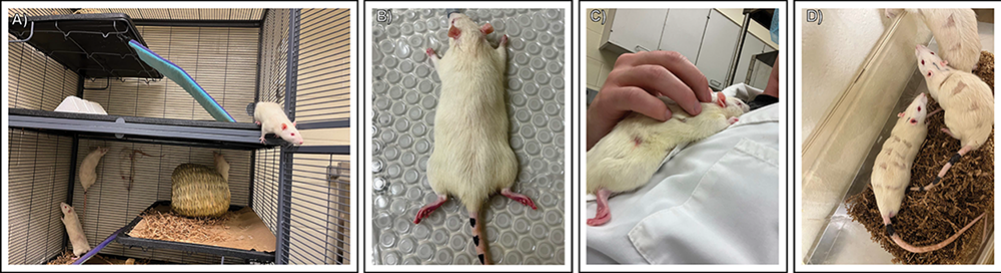
Subcutaneous insertion procedure for the hydrogel sensors. (A) Rats were permanently housed in sex-segregated cages with ample space for enrichment and exercise. Exercise wheels were later installed as they became available (B) Rats were lightly anesthetized with isoflurane gas; the implant areas were shaved and cleaned with an alcohol scrub before subcutaneous sensor insertion via an 18 gauge needle. (C) Rats were individually held to ensure warmth and comfort as they awakened after surgery. (D) Rats were temporarily housed in an observation cage before transfer back to their living space.

We aimed to evaluate the sensors under as near to real-world conditions as possible. To accomplish this goal, the rats were provided with ample space and encouraged to live an active lifestyle: they were housed in a large multistory cage (Critter Nation Double Unit) that provided 3 square feet of cage space per rat, with mezzanine levels and climbable walls. They were also provided with running wheels, bungalows, treats, and cardboard toys to play with (Figure 4A). Rats were fed and watered ad libitum and provided with wood blocks, cardboard huts, and paper for enrichment, along with regular treats consisting of dried mango, sunflower seeds, raisins, and Cheerios. Rats were socialized with researchers at least 3 times a week for at least 1 h upon arrival and throughout the course of the study. Rats were allowed to acclimate to the new facilities for at least 1 week before the insertion procedure. These conditions provide a much better living standard for the animals involved at little extra cost, while ensuring that the sensors were subjected to vigorous exercise and play to better model real-world use for patients.

At every procedure, every measurement time point, and every socialization time, each animal was carefully evaluated for health and welfare, and each sensor site for any clinical signs of rejection or infection. Evaluations were based on Texas A&M University guidelines on recognizing pain and distress in rats (TAMU-G-023) and evaluating each insertion site for clinical signs of inflammation. Subjects were scored on 5 criteria: Attitude & Posture, Gait & Movement, Surgical Sites, Appetite, and Elimination.

### 2.10 Insertion Procedure

Each rat was gently anesthetized in an inhalation box with oxygen (1 L/min) and isoflurane (flow setting 1–2), then transferred to the procedure area. The procedure area consisted of a nose cone with the same gas mixture, along with a clean heated water blanket held at a consistent 100 °F to maintain body temperature (Figure 4B). Rats were tested for pain response via gentle toe pinch, and breathing rate and coloration were carefully observed to maintain optimal anesthesia. Isoflurane flow settings were adjusted to optimize the response for each rat. Each eye was gently coated in petroleum jelly to keep the corneas hydrated during anesthesia.

Each rat had 12 potential insertion sites trimmed in a grid pattern along his back, in 3 columns of 4 rows (Figure S5). The insertion sites were brushed or vacuumed to clean stray fur and then cleaned with an alcohol solution. Insertion sites ranged from the shoulder blades to the flank. Of the 72 total potential sites, sensors were inserted at 48 (8 per sensor type, 8 sensors per animal). The remaining four positions on each rat were used for 1 positive control, 1 negative control, 1 sham control, and 1 parallel barcode experiment(Figure S6). Insertion sites were varied across each rat to ensure that each sample’s performance could be analyzed by position to address potential confounding results from different oxygenation levels and skin thicknesses between sensor positions (Figure S5). This ensured that each sensor type was represented in as many positions as possible. Shaved areas were kept to the minimum possible size to minimize postsurgical heat loss.

Sensors were carefully removed from their sterile containers and loaded into an Air-TITE 1 in. sterile 18-gauge cannula, then inserted subcutaneously using a sterile 18-gauge stainless steel rod. The cannula was withdrawn around the push rod, allowing the sensor to be gently deposited in the subcutaneous space. Direct, gentle pressure was applied to the skin above the insertion site for 30 s. Sensors were targeted to the dermis because of its relatively high water content and proximity to capillaries, which are ideal for allowing sensors to respond to environmental changes quickly.^25^

Barcode sensors were inserted using the same technique, using sterile 12- or 16-gauge 1 in. Air-TITE cannulas and appropriately sized push rods. Control solutions were inserted as 0.2 mL of solution directly from a syringe using a 20-gauge needle. The negative control sham injection sites were shaved identically to the other injection sites but received no injections.

After insertions were complete, each rat was given a unique tail marking, removed from anesthesia, and held in a researcher’s arms to warm each animal as he regained consciousness (Figure 4C). Each rat was evaluated for alertness and signs of distress before being placed back with cage mates(Figure 4D). Each rat was allowed at least 1 h recovery time before the first sensor readings.

### 2.11 Phosphorescence Lifetime Measurement Procedures

**Figure 5:**
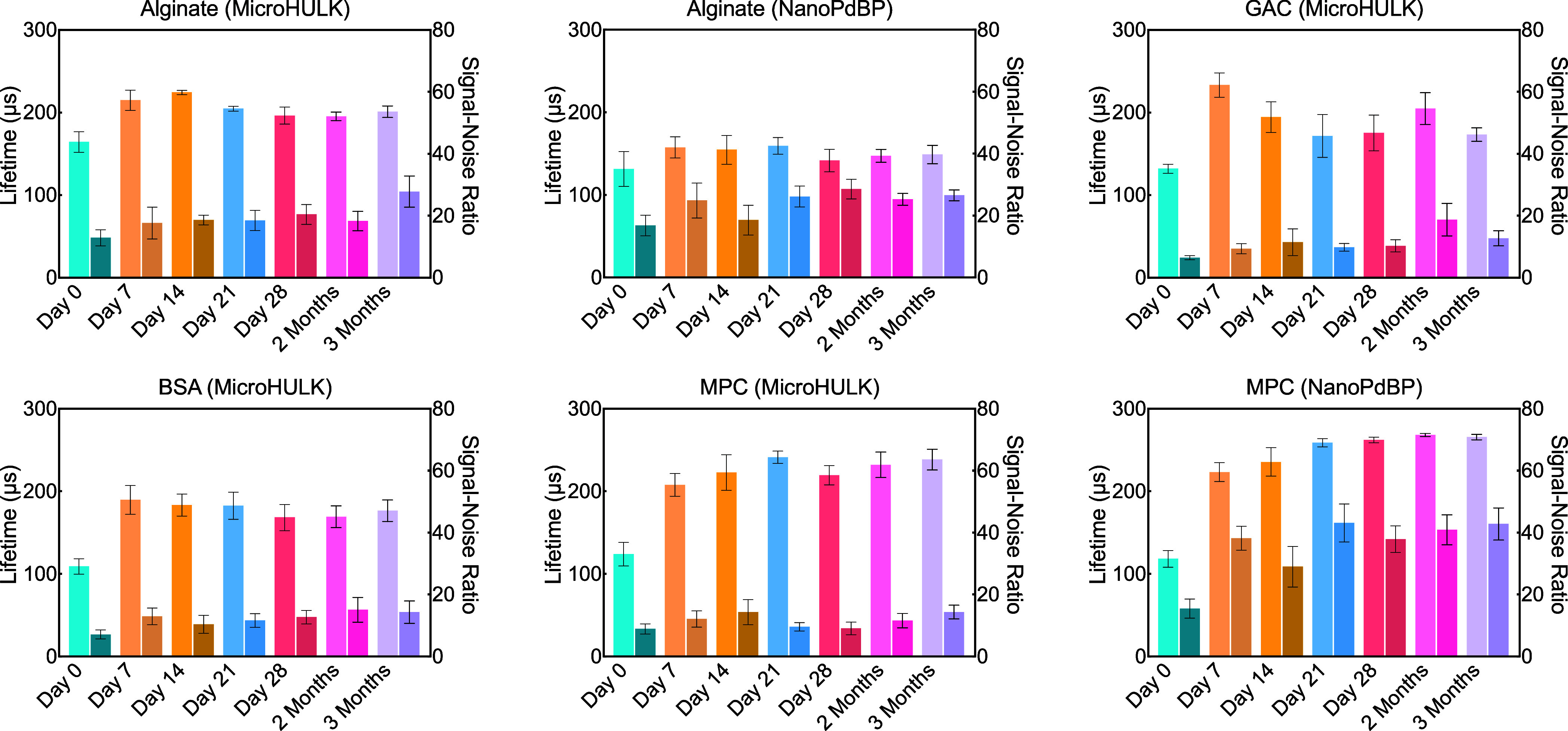
Primary sample lifetime and SNR. Averaged lifetimes are in microseconds for each material type tested. Signal to noise ratios are shown in the darker bars to the right of the primary bars. Results indicated that after the first day, lifetime data and signal strength retained consistent levels, particularly where signal strength was the strongest, and that the implants functioned *in vivo* as expected out to 3 months postimplantation.

**Figure 6:**
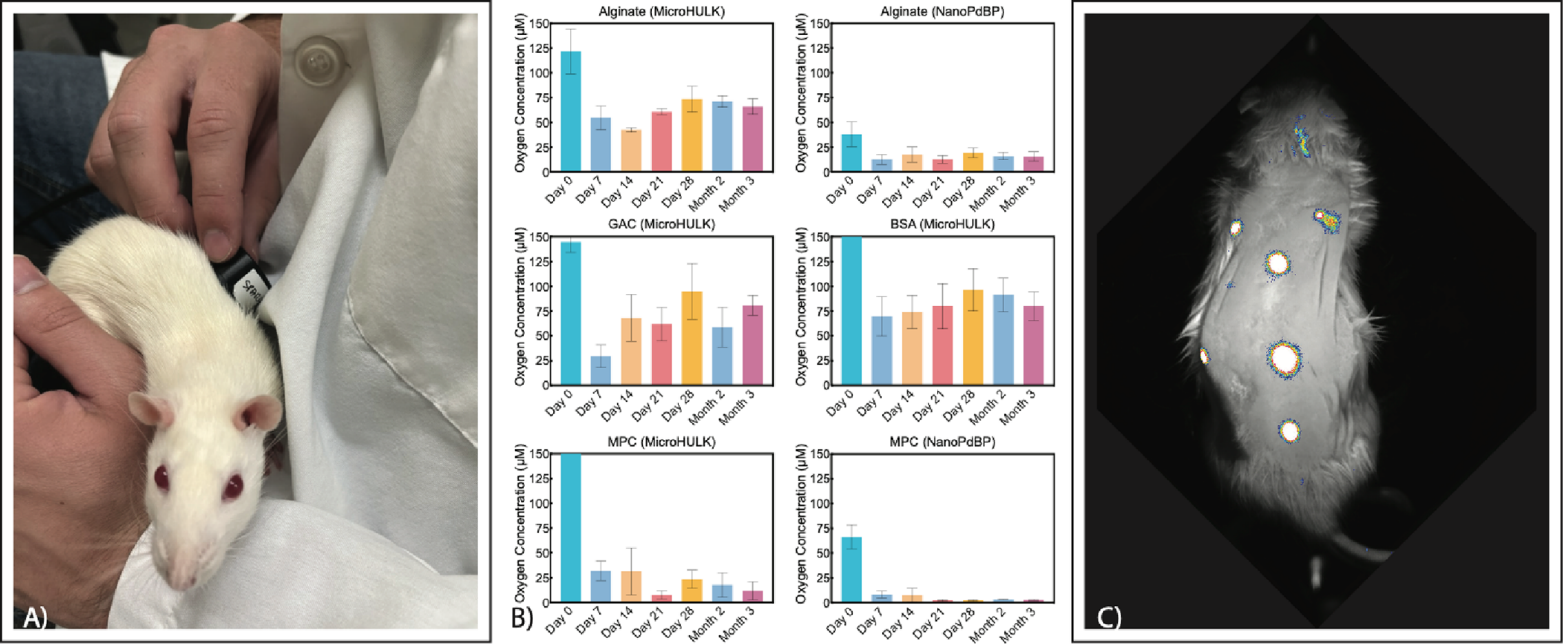
Results of in vivo oxygen evaluation. (A) Postsurgical oxygen monitoring of the implantable oxygen monitors. The reader heads are gently placed against the unrestrained rat’s skin. The excitatory red LED causes the phosphors to emit an infrared light for some microseconds. (B) Interstitial oxygen concentrations in micromoles, calculated by calibrating in vivo lifetime measurements to in vitro oxygen calibration data. (C) Qualitative visualization of the subcutaneous implants after 3 months in vivo, demonstrating the durability of the signal from implanted sensors.

Because of their extensive socialization, the rats were well acclimated to handling and satisfied to explore or sleep at their discretion when set on a researcher’s lap. This enabled regular readings to be taken without restraint or anesthesia of any kind. During readings, each rat was placed on the researcher’s lap and the reader head was gently held over each sensor site to collect lifetime readings (Figure 6A). Each sensor was read for at least 1 min to acquire at least 12 time points. If the reading was lost during measurement, then it was reestablished. The 10 time points with the best signal-to-noise ratio were averaged for each point and added and graphed for each separate sensor at each time point (Figure 5). Each animal was highly cooperative throughout the study and did not exhibit skittishness, even during the initial measurements. Oxygen concentration values (Figure6B) were calculated from calibrations using *in vitro* data for each material type, as described in section 2.7**.**

### 2.15 Fluorescence Imaging

After the scheduled 3 months of *in vivo* oxygen monitoring was finished, a fluorescent imaging system was used to visualize fluorescent intensity differences between sensors (Figure 6C). To maximize image clarity, each rat was briefly anesthetized and his back shaved. Fluorescence images were taken using an In Vivo Xtreme Optical imaging System (Bruker). No X-rays were used in this experiment. Rats were anesthetized with isoflurane, depilated, and then laid on the imaging window in the fluorescent imager. Excitation light was set to 630 nm, and emission light was collected at 790 nm for 5 s and overlaid with a reflectance background image. After imaging, rats were held in the researcher’s arms to keep warm until they regained consciousness and then returned to the transport cage.

### 2.16 Gross and Histology Processing

At the end of the study, rats were humanely euthanized with a sodium pentobarbital/phenytoin solution. A modified Rokitansky necropsy was used for the evaluation of all subjects in the study. Upon completion of the necropsy, the specimens were placed in 10% neutral buffered formalin. After initial fixation, sensors were explanted along with the surrounding tissue from each rat over the course of a week but remained in formalin prior to sectioning. Once fully fixed, an approximately 1 in. by 1 in. section of skin containing each sensor was removed; the sensor was transected perpendicularly to the long axis and submitted for histology. Due to the small size and transparency of the sensors, the reader head used to identify and obtain sensor signals *in vivo* was used at explant to help locate the sensors.

The necropsies, gross analysis, and histology processing and analysis were performed at the Cardiovascular Pathology Laboratory at Texas A&M University. Samples remained in formalin from 6 to 26 days post necropsy to adequately fix prior to sectioning. Tissues were processed, paraffin-embedded, sectioned, and stained according to the facility protocols. Tissues were processed, paraffin-embedded, sectioned, and stained with hematoxylin and eosin (H&E) and Masson’s Trichrome. Tissues were scored by a trained histopathologist for device presence, device zone, device state, host interface, and healing, as described in Table 1.

**Table 1:** Description of Metrics

**score**	**description**	**score**	**description**
**zone (layer of skin with a sensor)**		**host interface**	
1	epidermis	1	no reaction (smooth surface)
2	superficial dermis	2A	capsule, minimal
3	deep dermis	2B	capsule, mild
4	muscle layer	2C	capsule, moderate
5	submuscular	3A	infiltrative, peripheral
6	subcutis	3B	infiltrative, partial
		3C	infiltrative, diffuse
device presence			
Y1	yes, well-defined	cellular response (healing scale)	
Y2	yes, marginal	A	acute
N	no	SA	subacute
		C	chronic
device state		CP	chronic with phagocytosis
I	intact	CA	chronic-active
F	fragmented	CAP	chronic-active with phagocytosis
D	degraded	ACT	active
		R	remodeling
		H	healed

## 3 Results and Discussion

The purpose of this study was to evaluate the long-term performance and biocompatibility of our phosphorescence lifetime-based insertable oxygen sensor designs under real-world conditions, advancing a platform for developing a family of insertable metabolite sensors. In doing so, we engineered various designs by combining different biocompatible hydrogels with oxygen-sensitive phosphor micro- or nanocarriers. Each design was tested *in vitro*, *ex vivo*, and *in vivo* and then evaluated histologically. The results of each step provided valuable information for refining future insertable devices. Here, we discuss these results and their implications for future work. First, we discuss the overall results common to all tested devices, then assess the individual characteristics of each unique material combination.

### 3.1 *In Vitro* Results

*In vitro* results revealed fairly comparable oxygen sensitivity results among all HULK-microsphere based sensors, with Stern–Volmer constant (*K*_*SV*_) values ranging from 0.0069 to 0.0091 μM^–1^ depending on the hydrogel. In contrast, the oxygen sensitivities of PdBP-ECNP-based sensors in alginate and MPC hydrogel matrices were consistently and significantly higher, with *K*_*SV*_ values of 0.0227 and 0.02 μM^–1^, respectively. This elevated oxygen sensitivity may be attributed to the higher specific surface area of phosphor in the PdBP-ECNP system: ethylcellulose nanoparticles form a hydrophilic shell surrounding a hydrophobic core, which stabilizes the hydrophobic PdBP and discourages aggregation.^23^ In our earlier papers, microspheres have been used as the standard tool for enveloping oxygen sensitive phosphors because they can be used to localize enzymes adjacent to the phosphors and control diffusion rates. Fortunately, these approaches are not mutually exclusive, and these results suggest that the PdBP-ECNP system should be integrated into our microspheres in future studies (Figure S3).

### 3.2 *Ex Vivo* Results

The *ex vivo* test was designed to provide proof of concept in a realistic setting, to refine the design of the subsequent *in vivo* study by answering 3 key questions: Is the signal strong enough to read through the skin? Do the animals need to be shaved to read the sensors, or can the sensors be read through fur? Can the inserted sensors be reliably differentiated without interference from the other sensors?

Inserted sensor testing was initially performed through unshaved sites. Each sensor type was evaluated using the 1D reader heads, and a strong signal was found for each type, indicating that phosphorescence lifetime data could be reliably gathered through the rat dorsal skin. The results through the unshaved skin showed that all sensor types produced a strong signal that could be read through both skin and fur, indicating that shaving would not be necessary to read the sensors *in vivo*. Not shaving the insertion sites prevents shaving irritation from altering blood flow prior to the initial reading and also keeps the sensors at a more consistent temperature.

The final question answered by the *ex vivo* tests was one of positioning. The loose subcutaneous connective tissue in rats allows their skin to slide extensively over their body, which may improve flexibility in tight spaces and protect the skin from injury.^44^ However, this flexibility could be a liability for mapping subcutaneous sensors relative to the surface skin position. We tested sensors at a 1 in. spacing to determine whether their positions could be readily differentiated using the reader head, and found that sensors at this spacing could still be reliably differentiated from each other. Further, signals from neighboring insertion sites were shown to not interfere with each other during excitation and emission, which was expected from our previous modeling. In summary, these tests verified that each of the materials should be readable under both skin and fur in adult Sprague–Dawley rats and that the sensors would remain spatially well differentiated from each other after insertion. With this investigation complete, we moved on to the live test subjects.

### 3.3 *In Vivo* Results

For the acute phase of the foreign body response to the inserted devices, rapid changes in oxidation status can be expected to occur as an initial inflammation response to acute injury causes increased blood flow to the region. This acute response should subside quickly over a few days for biocompatible materials. Following the acute phase, the chronic phase involves gradual remodeling and potential capsule formation. With these healing processes in mind, we performed sensor readings at regular weekly intervals during the acute phase after insertion, starting on the day of the procedure and continuing to day 28. Thereafter in the chronic phase, readings were taken monthly. The overall trend in average lifetime values showed a lower lifetime value on the day of surgery followed by an increase by the next time point at day 7 that held steady over the next 3 months (Figure 5).

The subcutaneous tissue oxygen levels observed *in vivo* exhibited elevated oxygen concentrations on Day 0 (immediately after surgery) for all sensor types and then a drop to a lower concentration by Day 7 (Figure 6B). This course of events is expected because of the minor trauma from the cannula insertion, which causes local inflammation and blood vessel dilation, quickly followed by the recruitment of immune cells to the area. However, this acute reaction rapidly dissipates, dropping to a lower oxygen concentration by the second time point at Day 7, suggesting that the immune response is not sustained and may be muted by the minimally invasive insertion procedure and the biocompatible hydrogels chosen for the study.

From Day 7 through the end of the study at 3 months, oxygen levels remained relatively consistent for each hydrogel type. As expected from the varying material designs, different materials with varying chemical properties, the local oxygen concentration reported by each sensor did vary some among the different hydrogels as well as the embedded phosphor micro/nanocarrier.

While our experiment did not include direct comparison to other oxygenation metrics, we can compare our results with similar experiments published in the literature. In 2019, Profusa published a study measuring oxygen concentration using both transcutaneous oximetry and insertable oxygen sensors. Transcutaneous oximetry is a commercial method for assessing the oxygen diffusion across the skin. At baseline, transcutaneous oximetry in Kanick 2019 measured average values of 76 μM O_2_ with measurement ranging from 12 to 116 μM O_2_. Profusa’s insertable sensors measured interstitial oxygen concentrations ranging from 0 to 20 μM, with a median of 7.4 μM. Our Alginate NanoPdBP sensors likewise consistently reported 10–20 μM at least out to 3 months. MPC microHULK and MPC NanoPdBP reported similar ranges of 10–30 μM O_2_ and 0–9 μM O_2_, respectively. The other formulations, Alginate microHULK (50–70 μM O_2_); GAC microHULK (27–90 μM O_2_); and BSA microHULK (65–85 μM O_2_), reported somewhat higher but still reasonable values, which all fell comfortably between the ranges predicted by Profusa’s sensors and the values reported by transcutaneous oximetry.^12^

Based on this data, all formulations tested in our experiment reported realistic results that would be expected in this microenvironment according to available literature. We expect that the variations seen between different hydrogels and phosphor types may be the result of actual oxygen variations caused by the complex interactions between the unique microenvironments provided by each sensor type and the different host responses for each implant. We will investigate this exciting question further in future experiments.

One key finding from these tests is that the PdBP-in-ECNP sensors also showed consistently higher signal-to-noise ratios (SNRs) relative to HULK-in-microsphere sensors. This result is consistent with earlier *in vitro* tests, which showed that ethylcellulose nanofibrous networks stabilize the dispersion of PdBP phosphors by preventing hydrophobic phosphors from aggregating together into ineffectual clumps. This improved distribution of phosphors may improve sensitivity at low oxygen levels by maximizing oxygen interactions with phosphors.^23^ This effect may explain the lower oxygen levels reported in PdBP-in-ECNP sensors relative to those in HULK-in-microsphere versions. Overall, these results confirm that this effect does translate to a more intense signal *in vivo*, and further that this performance improvement remains consistent over the long-term *in vivo*.

#### 3.3.1 Fluorescent Imaging

Because sensors based on PdBP-in-ECNP produced consistently stronger intensities and higher signal-to-noise ratios relative to microsphere-encapsulated phosphors, fluorescence imaging could be used to visualize these differences as well as evaluate the sensors for migration over time. In agreement with the oxygen-monitoring data, fluorescence intensities were observed to be much higher for sensors with nanocomposite ethylcellulose-stabilized phosphors than for microsphere-encapsulated phosphors when compared directly. An example of these fluorescence images shows one subject’s sensors after 3 months (Figure 6C). Ethylcellulose containing sensors and PEG barcodes continued to fluoresce brightly under the skin, while most microsphere-encapsulated phosphors were more difficult to distinguish against background noise. In general, this higher intensity correlates with sensor signals that are easier to acquire. This test shows that the ethylcellulose stabilization continues to produce a stronger signal even after 3 months *in vivo*, despite using a smaller quantity of phosphor per unit volume of hydrogel. This suggests that ethylcellulose stabilization will be a valuable addition to phosphorescence lifetime sensors that will allow sensors to produce stronger signals and be read reliably even through thicker skin. Additionally, fluorescence imaging data showed no perceptible sensor migration from the insertion sites over the course of the 3-month study.

#### 3.3.2 Biocompatibility Assessment on Living Rats

Throughout the study, every welfare check showed that each rat was in good spirits, highly energetic, and sociable. Each study subject had consistently good body habitus with no guarding and the ravenous appetites expected of healthy young adult rats. The lack of any signs of inflammation or pain in the animals throughout the study is consistent with the hydrogel biomaterials selected for the sensors, which have suggested favorable biocompatibilities in the previous literature. Additionally, the lack of clinical signs of pain in the immediate aftermath of surgery is likely due to the soft, small form factor and the relatively innocuous insertion procedure, which required only the brief insertion of an 18-gauge needle at each site.

### 3.4 Histopathology Assessment

**Figure 7:**
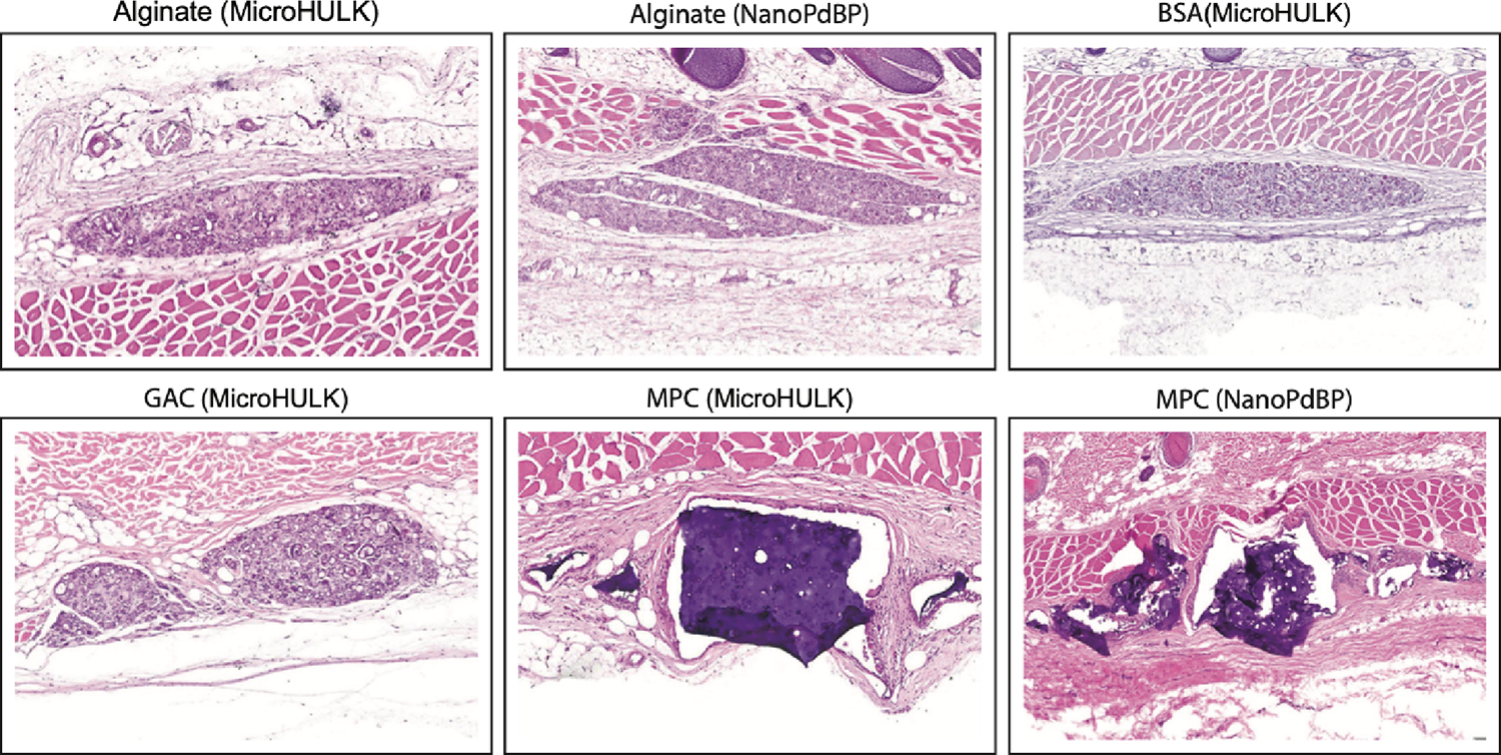
Summary of the sensors’ histological evaluation. Sensors were recovered from the animals at the conclusion of the study and stained with hematoxylin and eosin to evaluate the foreign body response. No elevated immune cell levels can be found within or around the implanted hydrogel, indicating that there is no detectable chronic inflammatory response to the insertable sensors. Hydrogels can be seen under different stages of degradation and cellular infiltration, as the implants are gradually broken down and removed from the body via phagocytosis, with no obvious effects on the local tissue.

**Figure 8:**
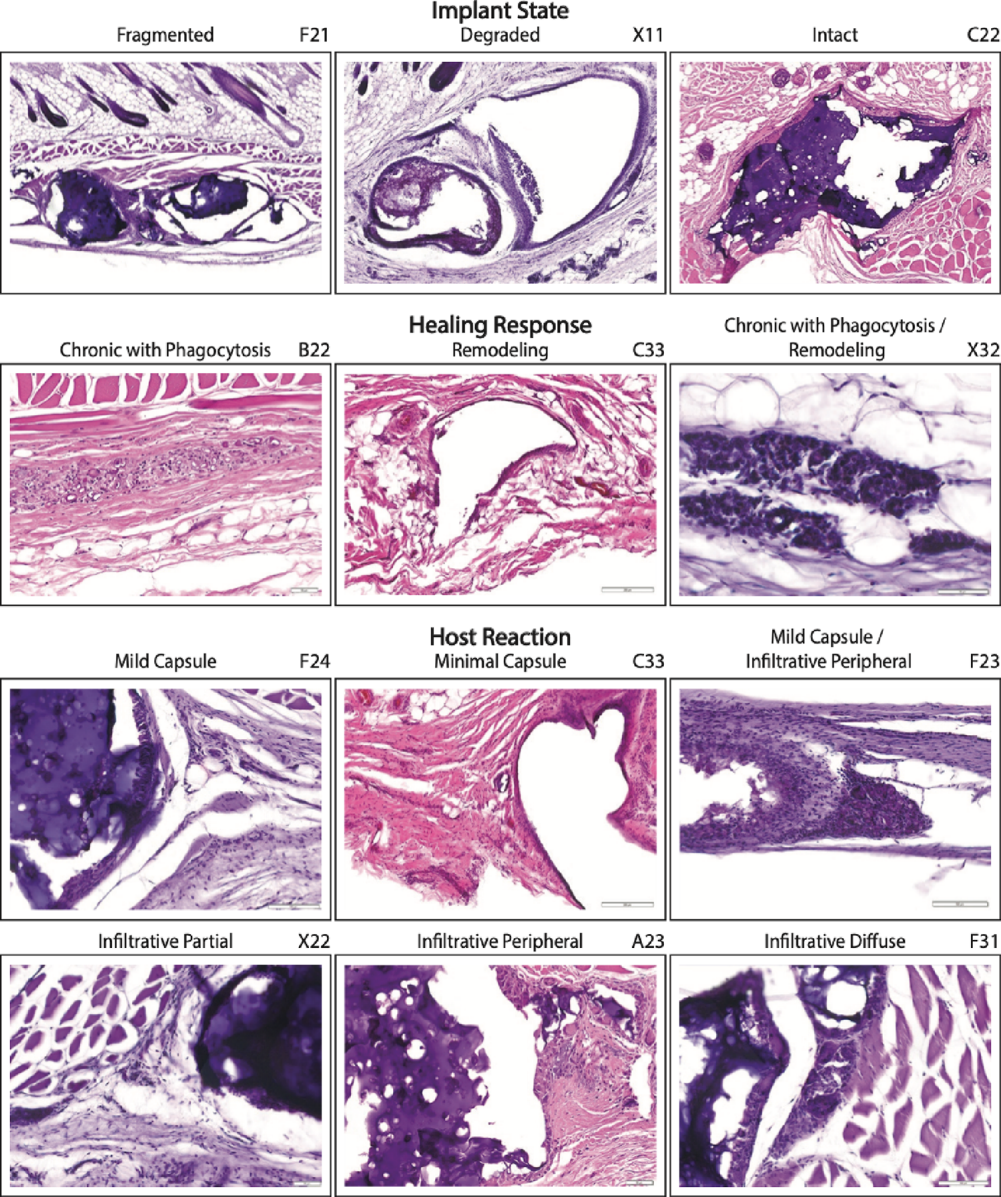
Primary response metrics. Images from recovered implants represent the range of primary response metrics observed in inserted sensors. The identity of each imaged sensor is indicated in the top right. All tested hydrogels exhibited good biocompatibility, so no images are available for rejection-type reactions like chronic-active response.

At the conclusion of the *in vivo* study, 41 of the 50 evaluated sensors were identified histologically. The inserted devices were uniformly recovered in the subcuticular zone between the deep dermis and the subcutis, and each sensor type had a distinct histological appearance (Figure 7). By the end of the experiment, most devices were noted to have some fragmentation, degradation, or a combination of both. Devices primarily exhibited pleomorphic, microfragmentation, and degraded particles along with diffuse infiltration of the sensor site by macrophages and multinucleated giant cells. Some of these macrophages and multinucleated giant cells contained intracytoplasmic fragments of device material. All insertion sites showed consistent signs of a continued healing host response around the devices. This healing response was noninflammatory in nature, and sensors exhibited minimal connective tissue encapsulation with gradual progressive removal of sensor material. The host–device interface was scored by classifying the response into one of three categories: no reaction, encapsulation, or an infiltrative response. All sensors exhibited some degree of encapsulation, infiltration, or both, (Figure 8). The evaluation metrics used by the blinded histopathologists and the scores for each are reported in Tables 1, 2, and 3.

**Table 2:** Zone and State Tallies by Sensor Type

		biosensor zone							state			
sensor type	epidermis	superficial dermis	deep dermis	deep dermis/muscle layer	muscle	muscle/submuscular	submuscular layer	subcutis	fragmented	intact	degraded	fragmented/degraded
BS (MicroHULK)							5/5		2/5		3/5	
alginate (MicroHULK)			1/7				5/7	1/7	3/7		2/7	2/7
GAC (MicroHULK)			1/6	1/6			1/6	3/6	1/6		4/6	1/6
MPC (MicroHULK)					1/5		3/5	1/5	3/5	2/5		
barcode					1/4		3/4		1/4	2/4	1/4	
alginate (NanoPdBP)					1/7	1/7	4/7	1/7	3/7		3/7	1/7
MPC (NanoPdBP)				1/7	2/7	1/7	2/7	1/7	3/7	4/7		

**Table 3:** Host Interface and Cellular Response by Sensor Type

	host interface										cellular response		
sensor type	no reaction/infiltrative diffuse	infiltrative peripheral	infiltrative partial	infiltrative diffuse	minimal capsule	mild capsule	minimal capsule/infiltrative peripheral	minimal capsule/infiltrative diffuse	mild capsule/infiltrative partial	mild capsule/infiltrative peripheral	chronic with phagocytosis	chronic with phagocytosis/remodeling	remodeling
BSA (MicroHULK)	1/5			4/5							5/5		
alginate (MicroHULK)	2/7			5/7							7/7		
GAC (MicroHULK)				5/6				1/6			5/6	1/6	
MPC (MicroHULK)			1/5	1/5		2/5	1/5				5/5		
barcode					2/4	1/4			1/4				4/4
alginate (NanoPdBP)								1/7	1/7		6/7		1/7
MPC (NanoPdBP)		2/7	1/7	1/7		1/7				2/7			

**Table 4:** Effect of E-Beam Irradiation on the Oxygen Sensitivity of Sensors in the Different Hydrogel Formulation

	**alginate (MicroHULK)**				**BSA (MicroHULK)**				**GAC (MicroHULK)**			
	**τ**_**0**_**± SD (μs)**	**τ ± SD at 257.9 μM O**_**2**_**(μs)**	*K*_*sv*_*(*μ**M**^**–1**^**)**	**% Δ (***K*_*sv*_*)*	**τ**_**0**_**± SD (μs)**	**τ ± SD at 257.9 μM O**_**2**_**(μs)**	*K*_*sv*_*(*μ**M**^**–1**^**)**	**% Δ (***K*_*sv*_*)*	**τ**_**0**_**± SD (μs)**	**τ ± SD at 257.9 μM O**_**2**_**(μs)**	*K*_*sv*_*(*μ**M**^**–1**^**)**	**% Δ (***K*_*sv*_*)*
control	246.42 ± 4.12	77.90 ± 4.41	0.0085		235.92 ± 2.04	70.19 ± 4.05	0.0093		259.75 ± 2.06	74.709 ± 5.27	0.0097	
E-beam	289.68 ± 2.50	100.62 ± 1.44	0.0069	21%	278.03 ± 1.36	90.79 ± 1.33	0.0078	18%	277.72 ± 1.76	90.90 ± 2.28	0.0078	22%
	**MPC (MicroHULK)**				**MPC (NanoPdBP)**				**alginate (NanoPdBP)**			
	**τ**_**0**_**± SD (μs)**	**τ ± SD at 257.9 μM O**_**2**_**(μs)**	*K*_*sv*_*(*μ**M**^**–1**^**)**	**% Δ (***K*_*sv*_*)*	**τ**_**0**_**± SD (μs)**	**τ ± SD at 257.9 μM O**_**2**_**(μs)**	*K*_*sv*_*(*μ**M**^**–1**^**)**	**% Δ (***K*_*sv*_*)*	**τ**_**0**_**± SD (μs)**	**τ ± SD at 257.9 μM O**_**2**_**(μs)**	*K*_*sv*_*(*μ**M**^**–1**^**)**	**% Δ (***K*_*sv*_*)*
control	265.47 ± 2.90	80.68 ± 0.45	0.0093		254.68 ± 3.03	42.52 ± 1.07	0.0189		245.32 ± 4.65	41.10 ± 0.66	0.0199	
E-beam	253.37 ± 15.04	77.36 ± 8.77	0.0091	2%	252.60 ± 4.97	42.19 ± 0.98	0.0200	6%	225.97 ± 6.70	34.72 ± 1.73	0.0227	13%

Taking these histological observations together with the data from our oxygen-sensitive sensor, the *in vivo* oxygen results match up closely with the expected physiological response of a quickly resolved minor trauma: initial oxygen concentrations were relatively elevated immediately after the insertion procedure due to local inflammation but had already subsided by the second measurement at 7 days and remained steady thereafter. This oxygen concentration is consistent with a pattern of insertion trauma followed by healing and degradation and further agreed with clinical observation of the rats, who revealed no signs of pain or inflammation following the initial insertion. Larger implantable biosensors have been reported to be less accurate for several days following implantation for the same reasons, so future experiments will include closer time points in the days following insertion to more precisely determine how quickly oxygen levels stabilize.

In this experiment, sensors were engineered by using hydrogels with different degradation rates. Protein-based gels (BSA and GAC) demonstrated the most degradation followed by polysaccharide-based alginate hydrogels, with synthetics (MPC and PEG) remaining intact or becoming fragmented with no observed degradation. The ideal degradation rate depends on the expected biosensor lifetime; therefore, this information is expected to be useful for tuning degradation rates in future insertable sensors.

The current regulatory landscape favors nondegradable devices, which can be removed after their useful lifetime to fully account for the inserted material. Nevertheless, degradable, insertable devices are a promising avenue for innovation because they do not require surgical implantation or removal. Instead, they can be inserted subcutaneously with a small needle and removed by the body itself over time. This spares patients from potentially invasive outpatient implantations and retrievals, while reducing procedure burdens on providers. Further, the quantity of palladium used in these insertable sensors is minute (less than 15 ng of Pd(II)) compared to the estimated 2000 ng/day average human palladium intake, primarily as Pd (II). Palladium is considered relatively safe among metal ions, and has an elimination half-life of only 5 to 12 days.^45^ More work is certainly warranted to establish the safety profile for this use case, as the degradation and clearance of these microspheres have not yet been evaluated. Nevertheless, the potential benefits of biodegradable sensors to patient well-being certainly merit future investigation.

### 3.5 Consistent Results Under Varied Conditions

Animal experiments reflect real-world use by accounting for variations at each insertion site. Rats, like humans, have natural variations in their skin. At different areas, skin may vary in thickness, fur density, looseness, and subcutaneous fat distribution, among other variables. Ideally, these natural differences should not significantly affect the sensor performance. To survey for possible positional effects, 12 locations were mapped to the dorsum on each rat, and sensors were assigned positions on each rat that put each sensor type in as many varied anatomical locations as possible (Figure S6). The results over the course of the study showed no significant variation based on any particular sensor position, and every position on the rats showed a good signal from the sensors. Anecdotally, the 3 sensors found deepest in the dermis (F13, S22, and X21) showed average to slightly above average SNR for their sensor types. Additionally, based on our *ex vivo* results showing strong signal strength through fur, the rats’ fur was allowed to regrow over the sensor sites over the course of the *in vivo* experiment. Fur regrowth did not interfere with the readability or signal strength.

This ability to maintain strong readouts also held when examining the device state and host reaction. No significant difference was seen between fragmented, degraded, and intact devices within each device type. This result can be explained by the phosphors’ oxygen sensitivity, which is independent of the device’s condition. As long as the phosphors remain in the local tissue environment, they continue to report the oxygen concentration. Similarly, the host response to the sensors did not produce a significant effect on the lifetime values or signal strength: neither encapsulation nor infiltration states correlated with signal strength. Due to the biocompatibility of the materials selected, most samples showed no capsule formation, and capsule material was typically seen alongside cellular infiltration in the same sample. Further, the observed encapsulation may be an artifact of inserting the sensors into an existing collagenous skin layer rather than a response to the devices. We expect that at longer time scales macrophages will remodel the degradable sensors enough to eventually cause a loss of signal strength, but that had not yet happened in any sensor type by the 3 month time point.

These results confirm that sensors yield overall reliable results that do not hinge on exact positioning or site condition. It also supports our previous findings from *in vitro* experiments, which showed that while apparent phosphorescence intensity depends on the intervening tissue, phosphorescence lifetime measurements are unaffected as long as the signal is strong enough to be reliably detected.^23,39^

### 3.6 Detailed Discussion by Sensor Type

While the sensor designs evaluated here share common underlying designs, each hydrogel and phosphor carrier possessed unique characteristics in terms of lifetime data, *in vivo* performance, and foreign body response. This follows naturally from the fact that the hydrogel carrier makes up the majority of the sensor and therefore plays a central role in both oxygen diffusion and *in vivo* interactions. Below, we discuss the results of the study specific to each hydrogel, focusing on hydrogel material properties, oxygen data and SNR over the course of the experiment, and specific biocompatibility results.

#### 3.6.1 Alginate with HULK-in-Microspheres

Alginate was selected as a hydrogel due to its successful track record as a biocompatible hydrogel *in vivo*. Alginate sensors can be fabricated using a simple and gentle process that results in robust mechanical properties that are well suited to handling during insertion. Alginate sensors with HULK-in-microspheres sensors reported a lifetime of 165 μs immediately after surgery, which we attribute to the higher blood flow and oxygen content caused by the inflammatory response to insertion. By week 1, however, the lifetime results had stabilized around 200 μs, and this level was maintained throughout the following 3 months of the study. SNR readings from these materials indicated a strong signal across the skin, with an initial SNR of 12 after surgery, followed by a steady level of 16–18 until the final time point. By calibrating these lifetime values with lifetimes at known oxygen concentration *in vitro*, the oxygen levels reported around the sensor at 120 μM of O_2_ immediately after insertion, which fell to around 50 μM at weeks 1 and 2, before gradually rising to about 70 um at 4 weeks, remaining steady thereafter for the next 2 months.

After recovery, alginate sensors were found primarily in the submuscularis zone, and all seven alginate hydrogels were at least fragmented, with two characterized as partially degraded and two as degraded. Interestingly, fragmentation state did not impact performance in any apparent way, either in reported lifetime or SNR. This result is not unexpected, as each oxygen sensing microsphere in the sensor works independently and does not require any specific arrangement to perform its function. The host reaction showed diffuse cellular infiltration throughout each sensor, with uniform chronic healing responses characterized by gradual phagocytosis of sensor material by macrophages and occasional multinucleated giant cells (MGCs), along with fibroblasts and collagen fibers. Macrophages and MGCs were observed enveloping both the alginate hydrogel and microparticles. This indicates that the alginate sensors were gradually biodegraded and remodeled over the course of the experiment without no evident chronic inflammation.

#### 3.6.2 Alginate with PdBP-in-ECNPs

Alginate with PdBP-in-ECNPs behaved broadly similarly to alginate with HULK-in-Microsphere sensors, but the few differences provided insight into the practical differences between the formulations *in vivo*. PdBP-in-ECNPs produced relatively lower lifetimes and higher SNR. From day 7 to 3 months, lifetimes averaged between 150 and 160 μs, roughly 50 μs shorter than standard alginate, while the average SNR was moderately higher, around 25. After calibration, these readings correlated to an initial O_2_ concentration of 36 μM on day 0, followed by O_2_ measurements between 10 and 20 μM for the course of the experiment. Both phosphor types fell within the expected oxygen concentration range for interstitial tissue, so both sensor groups may be accurately reporting a modest difference in local oxygen within the two sensors.^22^ The root of this difference will be explored in future studies.

The insertion zones, state, and healing were broadly similar to Alginate with HULK-in-Microspheres, which matches our expectation that the body’s response is driven primarily by the hydrogel. The only exception was that a single sensor was scored as mild capsule formation and only partial infiltration rather than diffuse, and one other exhibited possible minimal capsule formation. While this could indicate a minute shift in host response, this difference is more likely a result of the small sample size. Overall, the sensors were consistently biocompatible and noninflammatory and were gradually biodegraded by local phagocytes without a drop in performance.

#### 3.6.3 BSA with HULK-in-Microspheres

Bovine serum albumin (BSA)-based sensors were included to represent a protein-based hydrogel carrier for its good biocompatibility and enzymatic degradability *in vivo*. The lifetime data for BSA showed a lifetime of about 100 μs immediately after surgery, followed by lifetimes consistently averaging between 170 and 190 μs for the remainder of the experiment with SNR averaging from 10 to 15. Calibrated oxygen concentration after surgery was nearly 220 μM followed by concentrations between 65 and 85 μM over the next 3 months. Histologically, sensors were all found in the submuscular zone of the skin, with all 5 classified as at least fragmented and 3/5 as degraded. The host reaction and healing of BSA sensors were uniformly diffuse infiltration and chronic phagocytic degradation of the sensor material. BSA-based sensors overall exhibited good biocompatibility and consistency and are a promising candidate for exploring biodegradable nutrient sensors in future studies.

#### 3.6.4 GAC with HULK-in-Microspheres

Gelatin-alginate-collagen (GAC) is a hybrid of both polysaccharide and native extracellular protein-based materials, making it an intriguing candidate for evaluation because it combines the characteristics of both materials. These sensors averaged 132 μs lifetime immediately after insertion followed by lifetimes between 170 and 239 μs for the remainder of the 3 months. SNR levels averaged near 10 over the course of the experiment. Calibrated results revealed an initial oxygen level of 136 μM followed by an initially lower oxygen level of 27 μM on day 7. After day 14, oxygen levels averaged between 55 and 90 μM for the remaining part until 3 months.

Histologically, sensors were found in the deep dermis, deep dermis/muscle layer, submuscular layer, and subcutis. All 6 were classified as fragmented, degraded, or both. The host reaction and healing of GAC sensors were 5/6 diffuse infiltration and 1/6 initial capsule formation. All sensors showed a chronic phagocytic response, with one also showing additional signs of remodeling

It is reasonable to conclude that the higher oxygen levels seen in both the BSA and GAC sensors may be the result of the more rapid infiltration of cells and tissue seen throughout these protein-based sensors, resulting in extensive nutrient diffusion into these degradable sensors. The data here likewise support the continued exploration of GAC as a biomaterial for future degradable biosensors. In our future experiments, we intend to explore the different rates of biosensor infiltration and subsequent increased specific surface area on biosensor response times.

#### 3.6.5 MPC with HULK-in-Microspheres

MPC is a zwitterionic synthetic hydrogel based with antibiofouling properties that have been applied to cell culture equipment as well as contact lenses and stents.^36^ MPC sensors reported initial lifetimes near 125 μs immediately after insertion, followed by lifetimes between 205 and 240 μs for the duration of the study. SNR values ranged between 9 and 15. MPC sensors showed consistently lower oxygen concentrations relative to those of other hydrogel carriers. Initial calibrated oxygen values reported 146 μM oxygen immediately post surgery, after which the oxygen concentration ranged between 10 and 30 μM.

Histologically, 4/6 MPC sensors were primarily in the submuscular layer, with 1 in the muscle layer and 1 in the subcutis. 2/5 MPC gels were recovered intact, while 3/5 were fragmented. While all sensors showed chronic phagocytic healing, the host reactions varied more than other sensor types. Only 1 sensor had reached an infiltrative diffuse state by the end of the study, while the rest showed only peripheral infiltration or mild/minimal capsule formation. This indicates that the MPC hydrogel was more resistant to degradation *in vivo*, consistent with its synthetic chemistry, and limited host infiltration to the outer surface of the sensor.

#### 3.6.6 MPC with PdBP-in-ECNPs

MPC with PdBP-in-ECNPs phosphors reported a higher lifetime in MPC sensors relative to the HULK-in-microsphere formulation, with the initial lifetime reading at 120 μs followed by lifetimes ranging from 223 to 270 μs. SNR values were notably higher throughout the experiment, ranging from 29 to 43 over the course of the experiment. Calibrated oxygen concentration was recorded at 66 μM immediately after insertion followed by O_2_ levels ranging from 9 to 0 μM. This observed range is quite low, and could offer an additional explanation for the lack of cell presence throughout the material. The low oxygen concentration may be due to the host interface. MPC-based gels showed a greater propensity for mild capsule formation, and cellular infiltration into these hydrogels was mostly limited to the periphery of the sensor relative to the complete diffuse infiltrate observed in the other material types. The cases of infiltrative partial or diffuse host response may be an artifact of the friable nature of the MPC sensors, which were noticeably softer and more easily damaged than the other tested sensor materials.

## 4 Conclusions

In this study, we developed a family of oxygen-sensitive insertable hydrogel-based oxygen sensors based on the phosphorescence lifetime. These devices performed consistently for more than 3 months in an immune-competent animal model. Sensors reported interstitial oxygen concentrations in the physiological range throughout the experiment with different average oxygen concentrations between different hydrogel types and phosphor stabilization methods. Histopathological analysis confirmed that all designs were highly biocompatible and induced noninflammatory healing responses characterized by chronic phagocytic degradation of the devices. The degradation can even be tailored to specific future applications by choosing the appropriate hydrogel. Overall, we established the practical utility of insertable phosphorescence lifetime sensors, which can be made with a variety of hydrogel carriers and continue to function well over long-term implantation *in vivo*.

In our future work, this family of devices will be expanded to sense other important analytes by incorporating oxygen-depleting enzymes to create insertable biosensors for an array of clinically relevant metabolites including glucose and lactate. These insertable biosensors will be smaller and less obtrusive than existing wearable percutaneous and fully implantable biosensors, and we expect that they will represent an important new tool for both accessible healthcare and improving understanding of local metabolites in biomedical research.

## Supporting Information Available

S1: i*n vitro* calibration data of hydrogel sensors; S2: lifetime measurements comparison between ex vivo tissue and air; S3: microparticle characterizations using a cellometer, and images of hydrogel sensors; S4: reader head design and *in vitro* experimental setup; S5: lifetime and SNR data for control groups and secondary experimental series; S6: implant maps and distribution (PDF)
