# Supplementary material for: Long-Term Evaluation of Inserted Nanocomposite Hydrogel-Based Phosphorescent Oxygen Biosensors: Evolution of Local Tissue Oxygen Levels and Foreign Body Response
Source: ACS Appl Bio Mater. 2024 May 29;7(6):3964–80. doi: 10.1021/acsabm.4c00336 (PMC11190996; doi:10.1021/acsabm.4c00336)
Supplement: Supplementary file 1 — mt4c00336_si_001.pdf [file mt4c00336_si_001.pdf]

Supporting Information

Long-term Evaluation of Inserted Nanocomposite Hydrogel-based Phosphorescent Oxygen Biosensors: Evolution of Local Tissue Oxygen Levels and Foreign Body Response

David Chimene, Waqas Saleem, Nichole Longbottom, Brian Ko, Ananth Soundaram Jeevarathinam, Staci Horn, Michael J. McShane* E-mail: chimene@tamu.edu

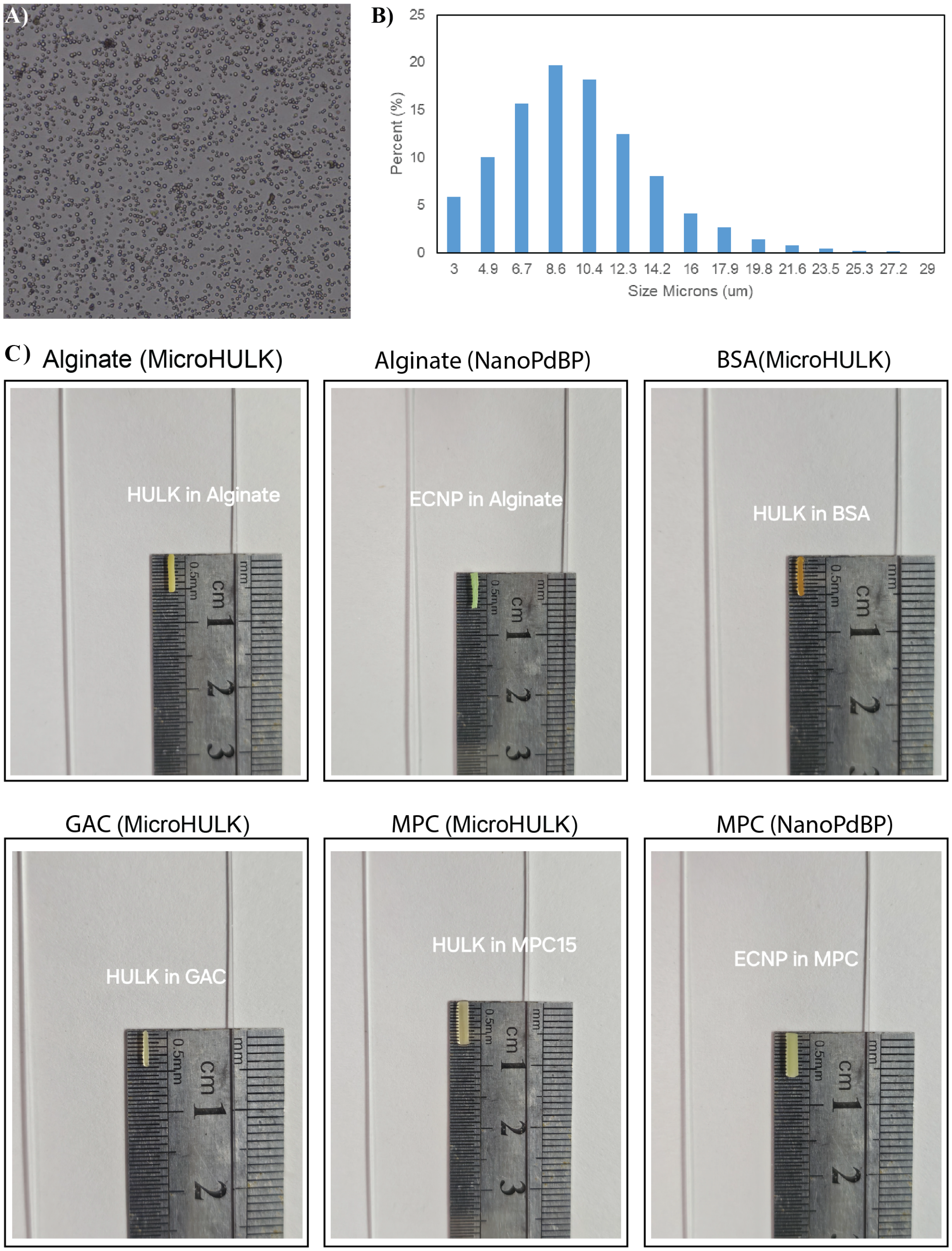

Figure S1: Microparticle Characterization and Sensor Measurements
(A) Microscopy of 1:50 diluted alginate microparticles in PSS wash solution ensuring minimal aggregation within the sample batch. **(B)** Histogram of alginate microparticle diameters following the layer-by-layer self-assembly procedure. **(C)** Sample images of each biosensor type. Each hydrogel oxygen sensor is approximately 5 mm long and 0.5 mm wide.

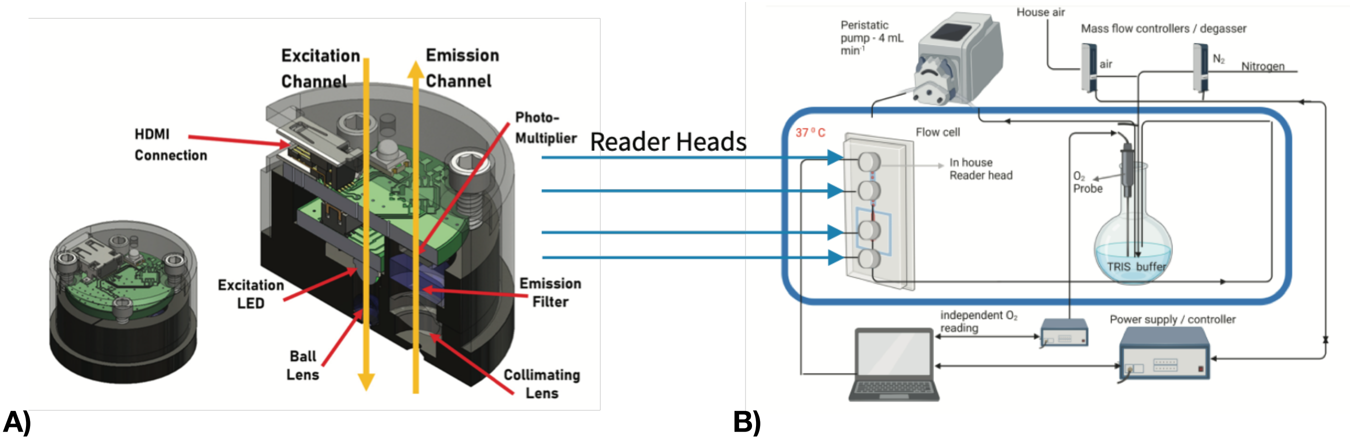

A)　　B)

Figure S2: Reader Head Design and In Vitro Experimental Setup
(A) A cutaway view of the reader head hardware used to quantify phosphorescence lifetime
(B) A schematic of the in vitro testing setup for evaluating implant lifetime under controlled oxygen conditions

*Subfigure A reproduced with permission from reference 39. Copyright 2022 IEEE

A. T. Zavareh, B. S. Ko, R. Horner, C. Lewis, M. J. McShane (2022) A One Inch in Diameter Point-of-Care Reader Head for the Measurement of Different Bio-Analytes Concentrations. in 2022 IEEE Healthcare Innovations and Point of Care Technologies (HI-POCT) (IEEE), pp 81-84.

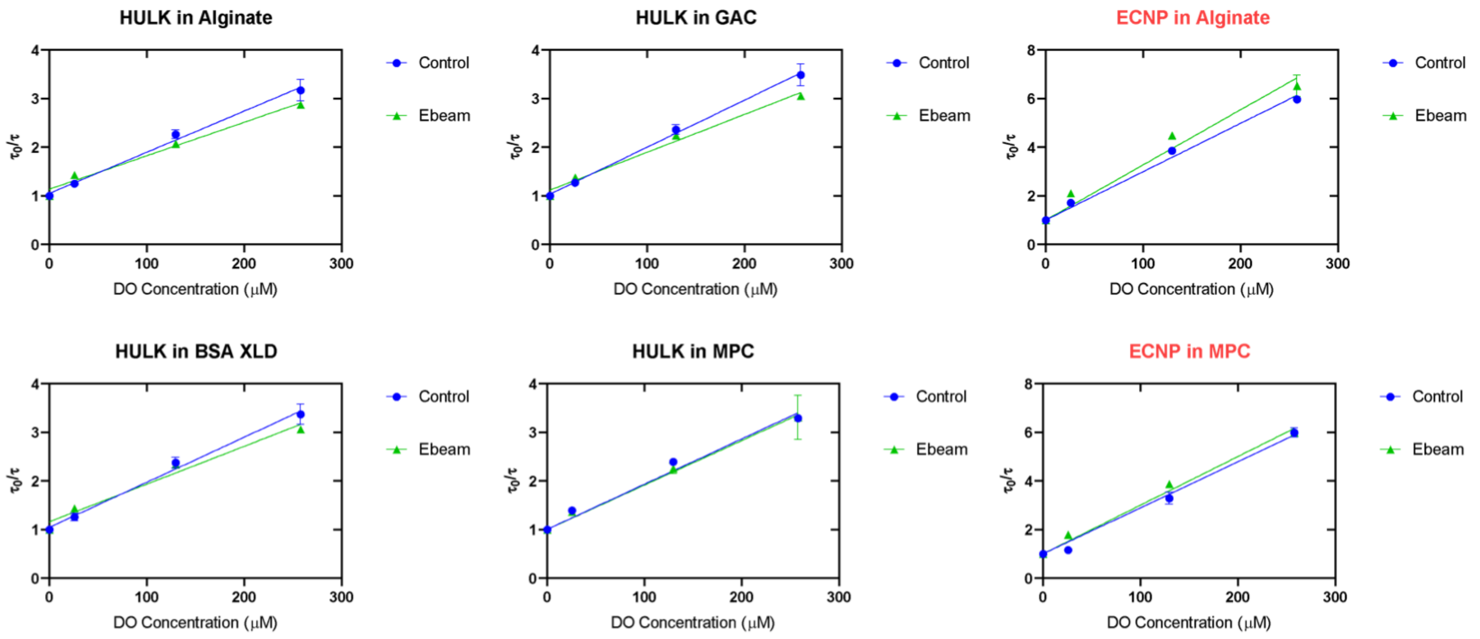

Figure S3: In Vitro Calibration Data
Overlaid Stern-Volmer plots from Control (N=3 or 4) and Ebeam irradiated (N=4) sensors. Using the in vitro experimental setup shown in Figure S4, each biosensor was exposed to known dissolved oxygen concentrations and lifetime data were collected, and Stern Volmer equations were generated. Later, these equations were used to translate in vivo lifetime measurements for calculating dissolved oxygen concentrations.

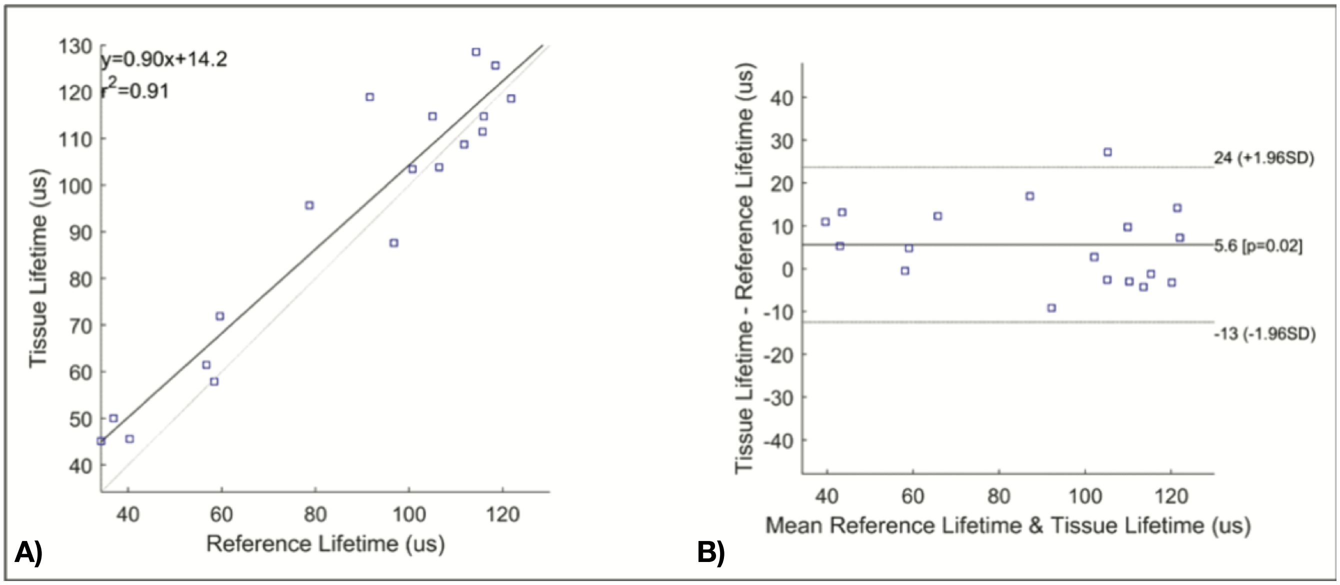

Figure S4
(A) A unity plot compares each sample's lifetime values obtained in room air to its lifetime value obtained in ex vivo testing after subcutaneous insertion. **(B)** A Bland-Altman plot summarizes the differences between tissue and reference lifetime values.

MALE 1 "Chief" (Left → Right; Cranial → Caudal)

	1	2	3
Cranial 1	Alginate (MicroHULK)	MPC (MicroHULK)	BSA (MicroHULK)
2	Silica Beads (Positive Control)	MPC (NanoPdBP)	Alginate (NanoPdBP)
3	Sterile Saline (Negative Control)	GAC (MicroHULK)	PEG (Barcode)
Caudal 4	MPC (MicroHULK)	No Injection (Sham Control)	Alginate (MicroHULK)

FEMALE 1 "Sweetheart"

	1	2	3
1	Alginate (MicroHULK)	No Injection (Sham Control)	MPC (MicroHULK)
2	PEG (Barcode)	GAC (MicroHULK)	Sterile Saline (Negative Control)
3	Alginate (NanoPdBP)	MPC (NanoPdBP)	Silica Beads (Positive Control)
4	BSA (MicroHULK)	MPC (MicroHULK)	Alginate (MicroHULK)

MALE 2 "Boss Man"

	1	2	3
1	Sterile Saline (Negative Control)	MPC (NanoPdBP)	Silica Beads (Positive Control)
2	GAC (MicroHULK)	Alginate (NanoPdBP)	Alginate (MicroHULK)
3	HEMA-CoA (Dual Strip)	No Injection (Sham Control)	BSA (MicroHULK)
4	MPC (NanoPdBP)	MPC (MicroHULK)	Alginate (NanoPdBP)

FEMALE 2 "Filips"

	1	2	3
1	Alginate (NanoPdBP)	MPC (MicroHULK)	MPC (NanoPdBP)
2	BSA (MicroHULK)	PEG (Barcode)	No Injection (Sham Control)
3	Alginate (MicroHULK)	Alginate (NanoPdBP)	GAC (MicroHULK)
4	Silica Beads (Positive Control)	MPC (NanoPdBP)	Sterile Saline (Negative Control)

MALE 3 "Ace"

	1	2	3
1	No Injection (Sham Control)	Alginate (MicroHULK)	MPC (MicroHULK)
2	Alginate (NanoPdBP)	Silica Beads (Positive Control)	BSA (MicroHULK)
3	GAC (MicroHULK)	MPC (NanoPdBP)	Sterile Saline (Negative Control)
4	BSA (MicroHULK)	GAC (MicroHULK)	HEMA-CoA (Dual Strip)

FEMALE 3 "Xena"

	1	2	3
1	PEG (Barcode)	GAC (MicroHULK)	BSA (MicroHULK)
2	Sterile Saline (Negative Control)	MPC (NanoPdBP)	GAC (MicroHULK)
3	BSA (MicroHULK)	Silica Beads (Positive Control)	No Injection (Sham Control)
4	MPC (MicroHULK)	Alginate (MicroHULK)	Alginate (NanoPdBP)

Sensor Description	n	Sensor Description	n
Alginate(MicroHULK)	8	HEMA-CoA (Dual Strip)	2
MPC (MicroHULK)	8	Barcode: PEG Matrix with varied enzymes	4
BSA(MicroHULK)	8	Silica Beads (Positive Control)	6
GAC(MicroHULK)	8	Sterile Saline (Negative Control)	6
Alginate (NanoPdBP)	8	No Injection (Sham Control)	6
MPC (NanoPdBP)	8	Total	72

Figure S5
Sensor maps were randomized for each rat to ensure that insertion site would not affect results. Total sensor numbers included 8 insertion sites for primary test materials as well as three control sites per rat.

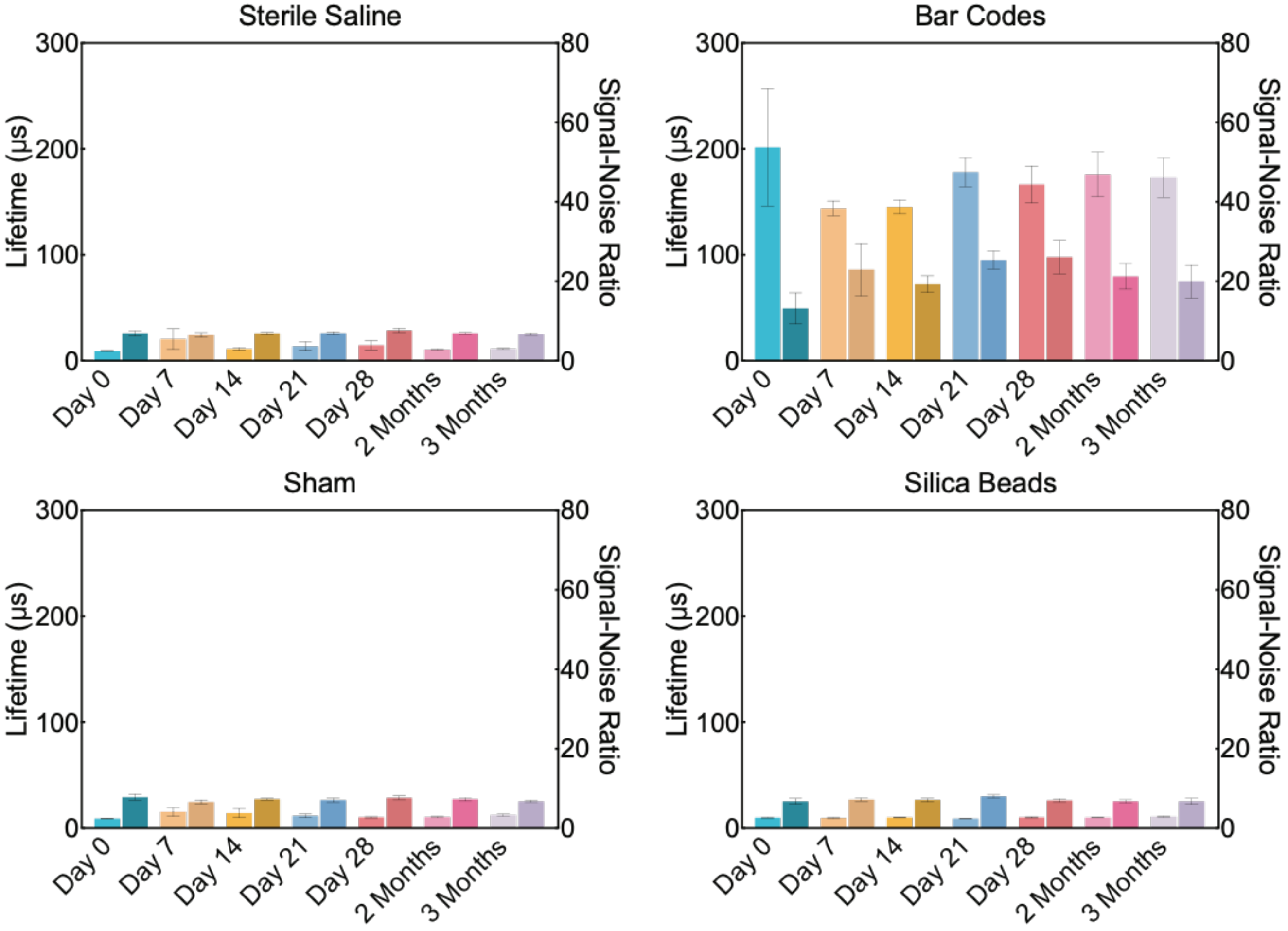

Figure S6
Lifetime and SNR Data for Controls and Secondary Experimental Series
